# A Propeller with a Flexible Twist: A Computational Analysis of Intrinsically Disordered Regions in PIEZO Gating and PIEZO-Associated Channelopathies

**DOI:** 10.3390/proteomes14030041

**Published:** 2026-08-11

**Authors:** Shivam Shukla, Mason Elzy, Abiral Shrestha, Vladimir N. Uversky

**Affiliations:** 1 Department of Integrative Biology, University of South Florida—St. Petersburg, St. Petersburg, FL 33701, USA; shivamshukla451@gmail.com; 2 Computational Biology Research Group, C. Leon King High School, Hillsborough County Public Schools, Tampa, FL 33610, USA; 7880660@hcps.net (M.E.); 1000020610@hcps.net (A.S.); 3 Department of Molecular Medicine and USF Health Byrd Alzheimer’s Research Institute, Morsani College of Medicine, University of South Florida, Tampa, FL 33612, USA

**Keywords:** mechanosensitive ion channel, PIEZO1, PIEZO2, channelopathy, intrinsically disordered region, liquid–liquid phase separation, protein–protein interaction, post-translational modification, proteoform

## Abstract

Background: Mechanosensitive ion channels PIEZO1 and PIEZO2 are key mediators of mechanotransduction, which converts physical forces into cellular signals involved in proprioception, touch, vascular function, and other physiological processes. Mutations in human PIEZO proteins are linked to various diseases, such as hereditary xerocytosis, lymphatic dysplasia, and proprioceptive dysfunction. However, the role of intrinsic disorder in the regulation of these proteins and their susceptibility for disease-associated mutations remains unclear. Methods: We analyzed canonical human PIEZO1 and PIEZO2 protein sequences using machine learning, neural network, and energy-based disorder predictors, together with the prediction of disorder-mediated binding regions, phase separation propensity, interaction networks, evolutionary conservation, clinically annotated human variants, and peptide structural modeling. Results: Both proteins showed moderate intrinsic disorder, with PIEZO2 having slightly greater disorder propensity and higher predicted phase separation potential. Intrinsically disordered regions frequently overlapped binding-prone segments and post-translational modification sites, supporting regulatory functions. Evolutionary comparisons showed strong conservation of PIEZO proteins, while selected disordered regions retained disorder propensity despite greater sequence variability. Disease-causing variants mainly affected the ordered regions of both proteins, whereas disordered regions contained proportionally more benign variants and relatively few pathogenic mutations. The modeling of mutations within disordered hotspots showed altered local conformational tendencies, indicating that some disease variants may disrupt dynamic interaction interfaces rather than global structure. Interaction network analysis linked both proteins to enriched mechanotransduction, ion transport, and cytoskeletal pathways. Conclusions: Overall, our findings identify intrinsic disorder as an underappreciated feature of PIEZO channel biology and provide a framework for interpreting PIEZO-associated channelopathies. PIEZO proteins also perfectly illustrate the proteoform concept, where one gene yields a highly diverse kit of mechanosensitive molecular tools. While humans only have two primary PIEZO genes (*PIEZO1* and *PIEZO2*), the body generates a vast array of functional variations.

## 1. Introduction

### 1.1. PIEZO Channels and Mechanotransduction

**Figure 1:**
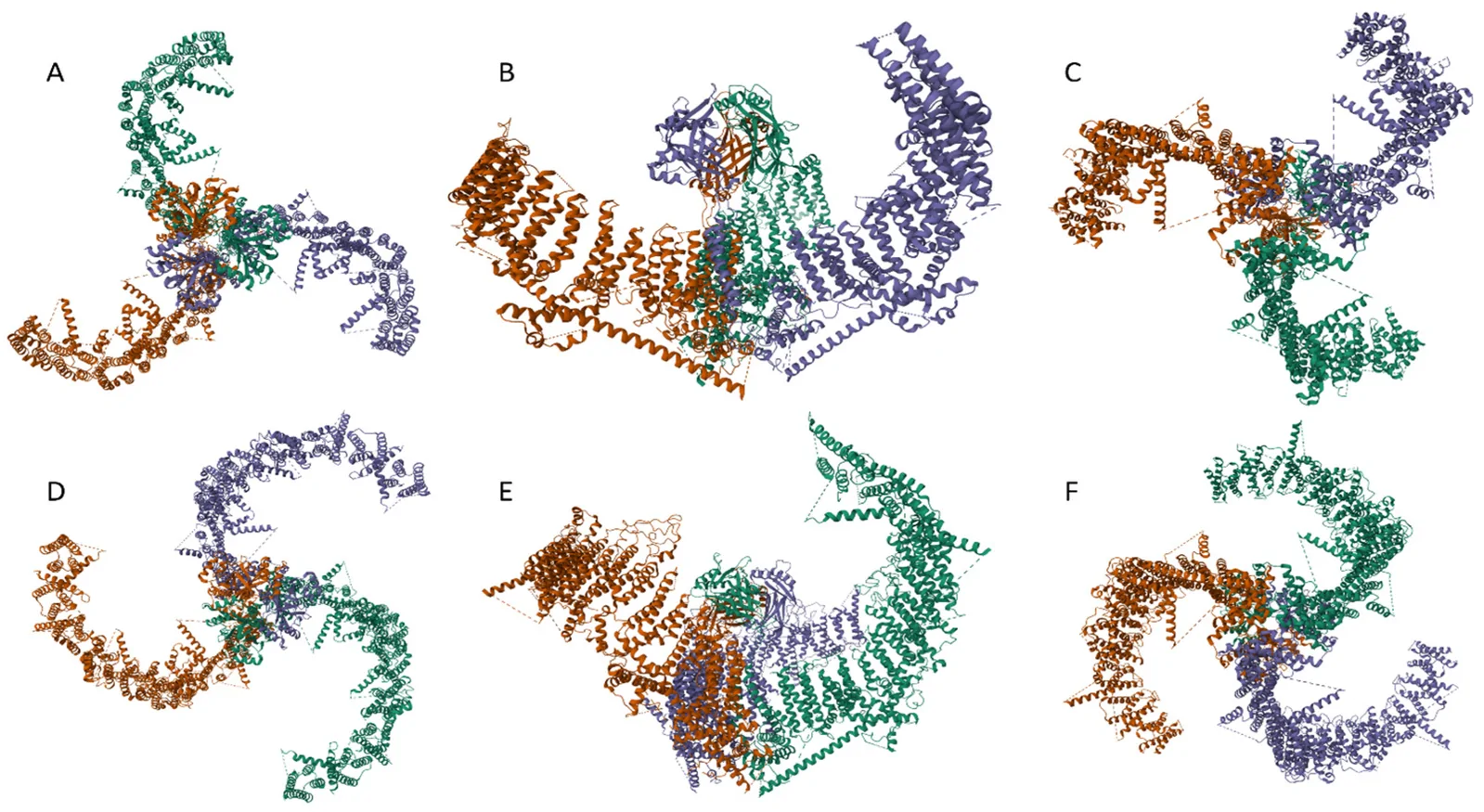
3D structures of the human mechanosensitive ion channel PIEZO1 ((**A**–**C**); PDB ID: 8YEZ; ref. [6]) and mouse PIEZO2 ((**D**–**F**); PDB ID: 6KG7; ref. [7]) determined by cryo-electron microscopy. Top, side, and bottom projections are shown in plots (**A**) and (**D**), (**B**) and (**E**), and (**C**) and (**F**), respectively. Structural information is not available for human PIEZO2; therefore, corresponding data were retrieved for its mouse homologue. Dashed lines correspond to missing electron density regions: residues 1–569, 645–677, 713–788, 880–914, 957–969, 1059–1995, 1122–1153, 1234–1282, 1371–1407, 1424–1512, 1569–1644, 1748–1778, 1804–1939, and 1981–2000 in PIEZO1 and residues 1–7, 143–200, 305–331, 359–490, 614–674, 824–933, 1383–1426, 1515–1556, 1578–1667, 1728–1947, 2056–2074, 2112–2235, and 2283–2298 in PIEZO2. The three PIEZO protomers are shown in orange, green, and purple for structural clarity.

Mechanosensation is a fundamental biological process where cells convert mechanical stimuli into biochemical and electrical signals. Central to this process are the mechanosensitive ion channels PIEZO1 and PIEZO2, which are large trimeric membrane proteins that mediate mechanically activated cation currents in response to membrane tension [1,2]. Named after the Greek term pίesi (πιέζω), meaning “to press” or “squeeze,” PIEZO channels act as specialized force sensors. They are defined by their ability to open and close in direct response to mechanical pressure, force, and membrane tension. These sophisticated molecular machines convert physical forces (like membrane tension, shear stress, and compression) into electrical or chemical signals. They are essential for diverse physiological processes, including vascular development, red blood cell volume regulation, and touch sensation. Specifically, PIEZO1 is widely expressed in non-neuronal tissues and plays a critical role in endothelial mechanotransduction, blood flow sensing, and erythrocyte volume regulation [3], whereas PIEZO2 is predominantly expressed in sensory neurons and is essential for touch and proprioception [4,5]. Structurally, PIEZO channels are massive, three-bladed-propeller-like structures that function as trimers embedded within the lipid bilayer (see Figure 1).

They have a central nonselective cation-permeable pore with a large-scale propeller-like assembly formed by three homomeric subunits [8], where curved blades deform the membrane and contribute to mechanosensitive gating [9,10,11,12]. Structure-guided research has unveiled how specific domains, individual amino acids, and interdomain interactions govern key functional properties of PIEZO channels, such as ion permeation, inactivation, voltage gating, and response to modulators [7,13,14,15,16,17,18,19,20,21,22,23]. In the PIEZO1 structure, there are large beams that span from the central pore to the edge of protein and act as levers, transmitting peripheral forces to the gate [10,12]. Concurrently, PIEZO1 operates via a force-from-lipids mechanism, where membrane forces induce conformational changes [24,25,26,27,28]. Therefore, proper PIEZO function necessitates a combination of specific lipid interactions and broader membrane physical changes [22,29,30]. This conclusion is supported by the observation that PIEZO1 activity is directly regulated by the membrane lipid makeup and the arrangement of cholesterol-rich domains, which organize and coordinate the function of the clustered channel, as evidenced by the fact that the manipulation of membrane cholesterol alters PIEZO1-mediated mechanotransduction [30]. Mutations in PIEZO1 are associated with various human diseases, including hereditary xerocytosis and lymphatic dysplasia, while PIEZO2 mutations cause maladies affecting touch perception and proprioception [31,32]. Despite advances in structural and functional characterization, the molecular determinants that separate regulatory regions from disease-sensitive regions within PIEZO proteins remain incompletely understood.

### 1.2. Intrinsic Disorder in Proteins

Intrinsically disordered proteins (IDPs) and intrinsically disordered regions (IDRs) lack a stable tertiary structure under physiological conditions yet can play critical roles in cellular functions, like regulation and signaling [33,34]. Unlike structured proteins, IDRs exist as dynamic conformational ensembles that enable functional versatility, including transient interactions, post-translational modification (PTM) integration, and molecular recognition through disorder-to-order transitions.

A defining feature of IDRs is their enrichment in polar and charged residues and depletion of hydrophobic amino acids, which prevents stable folding while promoting flexibility [35]. This structural plasticity allows IDRs to function as hubs in protein–protein interaction networks and to mediate context-dependent binding through short, linear motifs or molecular recognition features (MoRFs) [36]. Research, both computational and experimental, has revealed that intrinsic disorder is widespread across proteomes and is particularly enriched in regulatory and signaling proteins [33,37].

Importantly, the structure of proteins, even ordered ones, represents a dynamic conformational “mosaic” rather than static blocks [36,38]. Because different parts fold to varying degrees and constantly shift between folded and unfolded states, proteins form a flexible “ensemble” of structures [39,40,41]. This expands the structural and functional diversity of proteins and directly aligns with the proteoform and structure–function continuum concepts. In this framework, a single gene produces various distinct molecular forms (proteoforms). These diverse forms coexist as a highly dynamic pool of different shapes and functions [38,42,43].

### 1.3. Intrinsic Disorder, Liquid–Liquid Phase Separation, and Biomolecular Condensates

It is accepted now that along with traditional membrane-bound organelles, intracellular space features various membrane-less compartments known as membrane-less organelles (MLOs) or biomolecular condensates (BCs) that selectively compartmentalize proteins and nucleic acids, thereby facilitating a wide array of functions [44,45,46,47]. Since MLOs/BCs lack a surrounding lipid membrane, their constituents constantly partition and diffuse between the dense droplet phase and the surrounding cytoplasm or nucleoplasm [48,49,50]. These highly responsive, dynamic cellular compartments are characterized by liquid-like properties and are capable of spontaneous formation and dissipation [51,52,53,54,55,56,57,58,59,60,61]. Being originally considered a specific feature of eukaryotic cells, MLOs/BCs are increasingly recognized as crucial organizers of various cellular processes in all domains of life—Bacteria, Archaea, and Eukaryota [62,63,64,65,66,67,68]—and were even shown to contribute to viral replication cycles [69,70,71,72].

MLOs/BCs are formed via liquid–liquid phase separation (LLPS), the physical process that builds these compartments within the cell. In the context of intracellular LLPS, the two “liquids” refer to a dense protein-rich phase (droplets) and a dilute protein-poor phase, both suspended in the aqueous intracellular media. The dense phase remains fluid, allowing rapid internal diffusion of molecules, rather than aggregating into a solid or crystalline state. These phase transitions are physically driven by weak, multivalent, transient interactions, such as electrostatic, hydrophobic, π-π stacking, and electrostatic–π interactions, enabling rapid binding and release cycles, which are encoded within the amino acid sequence of the intrinsically disordered regions. Therefore, due to their high structural flexibility, binding promiscuity, and ability to be involved in weak polyvalent interactions, IDPs are commonly recognized as important players in the biogenesis of MLOs and BCs [67,73,74,75,76,77]. In fact, IDPs act as the primary drivers of LLPS [57,78,79,80,81,82,83,84,85], as the lack of a rigid 3D structure allows IDPs to behave like flexible strings, using highly dynamic and weak interactions to recruit other molecules and create concentrated droplet-like compartments within the cell.

### 1.4. Intrinsic Disorder and Disease

Intrinsic disorder plays an important role in many human diseases because IDRs frequently mediate signaling, regulation, and protein–protein interactions that are sensitive to sequence perturbation. Dysregulation of IDPs has been associated with cancer, neurodegeneration, cardiovascular disease, and developmental disorders [86,87]. For example, the tumor suppressor p53 contains extensive disordered regions required for transcriptional regulation and partner binding, and mutations affecting p53 function are among the most common events in human cancers [88]. Similarly, α-synuclein, a largely intrinsically disordered protein, is strongly linked to Parkinson’s disease through aggregation-prone conformational transitions and pathogenic mutations [89,90].

Disease-associated mutations often affect structured and disordered regions through different mechanisms. Mutations in ordered regions commonly disrupt folding, stability, catalytic activity, or ion conduction, whereas mutations in IDRs more often alter binding affinity, interaction specificity, post-translational regulation, or conformation [91,92]. This has been observed across many disease systems, where structured regions in proteins tend to mediate core biochemical activity while disordered regions function as regulatory interfaces. This means that mutations in IDRs may produce disease phenotypes without causing large-scale structural destabilization.

Recent studies have shown that IDRs frequently serve as disease-relevant hotspots because they integrate signaling inputs and organize transient interaction networks that are needed for normal cellular homeostasis [87,93]. However, the role that intrinsic disorder plays in disease mechanisms remains understudied in large membrane proteins, particularly ion channels like PIEZO1 and PIEZO2. Mutations in PIEZO1 and PIEZO2 have been associated with multiple diseases related to vascular development, red blood cell hydration, and sensory perception, such as dehydrated hereditary stomatocytosis (also known as hereditary xerocytosis), generalized lymphatic dysplasia (GLD), bicuspid aortic valve (BAV), distal arthrogryposis (type 3 (Gordon syndrome), type 5, and Marden-Walker Syndrome), and sensory dysfunction (e.g., distal sensory neuropathy) [1,31,32]. Furthermore, beyond direct genetic mutations, PIEZO channel dysfunction (which often originates from altered activity rather than inherited mutations) is implicated in a broad range of pathologies, notably in neurological disorders (Alzheimer’s disease, multiple sclerosis, and stroke), ocular diseases (glaucoma, myopia, cataracts), inflammatory bowel disease, and cancer, where PIEZO1 drives tumor growth and metastasis [94,95,96,97,98,99,100,101]. Furthermore, it was hypothesized that delayed-onset muscle soreness (DOMS) and post-orgasmic illness syndrome (POIS) represent a form of the acquired PIEZO2 channelopathy associated with the acute mechanical stress-derived neuronal energy-depleted impairment of PIEZO2 channels at the proprioceptive sensory terminals [102]. Furthermore, it was hypothesized that the acquired PIEZO2 channelopathy-induced proton reversal of glutamatergic somatosensory terminals may constitute the primary initiating switch mechanism that drives the pathogenic derailment of liquid–liquid phase separation (LLPS) underlying condensopathies, such as cancer, inflammation, virus infection, and neurodegenerative diseases, including amyotrophic lateral sclerosis (ALS) [103]. Determining how intrinsic disorder relates to these disease-associated mutations in PIEZO channels can provide new insight into channel regulation and channelopathy pathogenesis.

### 1.5. Objectives

In this study, we aim to determine whether intrinsic disorder contributes to the structural regulation and disease-associated mutational landscape of the mechanosensitive ion channels PIEZO1 and PIEZO2. Specifically, we investigated whether these large membrane proteins contain IDRs, whether such regions overlap with predicted interaction-prone segments, whether disease-associated mutations are distributed differently between ordered and disordered regions, and whether selected mutations alter local disorder propensity or peptide structure. By combining machine learning, neural network, and energy-based prediction models, evolutionary conservation analysis, experimental human variant and mutation data, mutant peptide modeling, and statistical analysis, we aimed to determine whether intrinsic disorder represents an unknown component of PIEZO channels and whether it may help explain mechanisms underlying PIEZO-associated disease.

## 2. Materials and Methods

### 2.1. Protein Sequence Retrieval and Dataset Construction

Protein sequence data for the mechanosensitive ion channels PIEZO1 and PIEZO2 were obtained from the UniProt Consortium database (uniprot.org) [104]. Canonical human protein sequences were selected to ensure consistency for all downstream analyses and to avoid redundancy associated with isoform variation.

The reviewed (SwissProt) entries for human PIEZO1 (PIEZO-type mechanosensitive ion channel component 1; UniProt ID: Q92508) and PIEZO2 (PIEZO-type mechanosensitive ion channel component 2; UniProt ID: Q9H5I5) were used. Full-length amino acid sequences were downloaded in FASTA format and used for all intrinsic disorder, interaction, mutation, and phase separation analyses. Additionally, a UniProt feature annotation table for each protein was downloaded and used to analyze sequence features, including transmembrane helices, topological domains, compositional bias regions, modified residues (post-translational modifications), natural variants, and other experimentally or computationally determined functional elements.

### 2.2. Intrinsic Disorder Prediction and Quantification

Intrinsic disorder within the full-length protein sequences of PIEZO1 and PIEZO2 was evaluated using a multi-predictor approach through the RIDAO platform (version 2026.6.20). This approach integrates several disorder prediction algorithms to model intrinsic disorder propensity across protein sequences.

The predictors used in this study, PONDR^®^ VLXT, PONDR^®^ VSL2, PONDR^®^ VL3, IUPred-S, IUPred-L, and PONDR^®^ FIT, are based on a combination of computational models, including machine learning, artificial intelligence, neural network-based classifiers, and physicochemical energy estimation models [105,106,107,108,109]. Neural network-based predictors such as the PONDR family are trained on experimentally validated datasets of ordered and disordered proteins, while tools like IUPred estimate intrinsic disorder based on the inability of residues to form stabilizing inter-residue interactions. Lastly, PONDR-FIT is a meta-predictor that integrates multiple algorithms to predict disorder.

For each predictor, per-residue disorder scores ranging from 0 to 1 were generated, where scores above 0.5 indicate a high likelihood of intrinsic disorder [108,109]. Two primary metrics were calculated from these outputs: (1) the percentage of predicted intrinsically disordered residues (PPIDR), which is the proportion of residues with disorder scores >0.5, and (2) the average disorder score (ADS), which is the average disorder propensity across the entire protein sequence. Two simple linear regressions were performed in R for PIEZO1 and PIEZO2 to test the relationship between PPIDR and ADS across prediction algorithms.

In addition to individual model outputs, the mean disorder profile (MDP) was calculated as the average disorder score across all predictors for each residue position. This consensus-based approach reduces predictor-specific bias and provides a more reliable estimate of intrinsic disorder [33,109].

Furthermore, disorder-based binding regions were predicted using ANCHOR2. ANCHOR2 identifies segments within the protein’s disordered regions that are likely to undergo disorder-to-order transitions upon binding to structured partners [110,111,112]. These regions, called molecular recognition features (MoRFs), are characterized by favorable interaction potential despite lacking stable structure in isolation. Residues with ANCHOR scores > 0.5 were classified as putative binding regions.

### 2.3. Evaluation of the Liquid–Liquid Phase Separation Propensity

The probability of PIEZO1 and PIEZO2 to undergo liquid–liquid phase separation (LLPS) was evaluated using the FuzDrop algorithm (fuzdrop.bio.unipd.it) [113,114,115]. FuzDrop predicts spontaneous droplet formation from amino acid sequence based on features associated with intrinsic disorder, multivalent interactions, and aggregation propensity. FuzDrop predicts sequence-based LLPS propensity rather than directly measuring phase separation. Phase separation can result from multivalent interactions that promote the formation of protein-rich and protein-poor phases under suitable cellular conditions. MolPhase (molphase.sbs.ntu.edu.sg), an algorithm that uses physicochemical features and experimental data to predict protein phase separation, was also used for this analysis. The predictors utilized in this study (FuzDrop and MolPhase [116]) do not just arbitrarily label sequences. Instead, they calculate specific biophysical parameters, such as local sequence pattern asymmetry, hydropathy, and net charge, which have been experimentally validated in the literature to correlate with a protein’s propensity to undergo phase separation. Therefore, these tools estimate this propensity but do not model thermodynamics or phase boundaries.

Full-length protein sequences were used to obtain the probability of spontaneous phase separation (p_LLPS_), residue-level droplet-promoting probabilities (p_DP_), predicted droplet-promoting regions (DPRs, i.e., regions containing at least five consecutive residues with p_DP_ ≥ 0.60), aggregation hotspots (i.e., regions containing droplet-promoting residues (p_DP_ ≥ 0.60) and exhibiting interaction mode divergence (S_bind_ ≥ 2.2), making them sensitive to the cellular context), and cellular context-dependent interaction regions (S_bind_), which identify residues predicted to participate in context-dependent interactions that may promote or regulate biomolecular condensate formation. According to the established FuzDrop framework [113,114,115], the relation of a protein to LLPS can be computationally evaluated from its amino acid sequence based on the p_LLPS_ value and DRP information. Here, proteins with p_LLPS_ ≥ 0.60 are classified as predicted droplet drivers (i.e., proteins capable of undergoing spontaneous LLPS), DPR-containing proteins with p_LLPS_ < 0.60 are viewed as droplet clients (i.e., proteins capable of partitioning into droplets/condensates), whereas the DPR-less proteins with p_LLPS_ < 0.60 are not considered to be related to LLPS processes [113,114,115]. These outputs were analyzed alongside intrinsic disorder and variant data to evaluate whether disordered regions of PIEZO channels contribute to dynamic higher-order assemblies.

### 2.4. Protein–Protein Interaction Network Analysis and Gene Ontology

Protein–protein interaction (PPI) networks for PIEZO1 and PIEZO2 were generated using the STRING database (version 12.0) [117]. STRING integrates known and predicted functional associations derived from experimental data, curated databases, co-expression, and computational prediction methods rather than providing precise structural or thermodynamic data.

Individual protein networks were constructed using *Homo sapiens* as the reference organism with a minimum interaction score of 0.7 (high confidence). STRING confidence scores range from 0 to 1 and reflect the confidence that a functional association is supported by the available evidence, rather than the physical strength of an interaction. All available interaction sources were included. Network metrics, including node and edge counts and average node degree, were recorded to understand network complexity. Protein–protein interaction enrichment was also evaluated. Next, a joint PPI interactome of PIEZO1 and PIEZO2 was created (medium confidence; 0.4 minimum interaction score) and analyzed using similar metrics as above. This less restrictive threshold was selected to capture a broader shared interaction network for exploratory analysis while still excluding low-confidence associations.

Proteins in these networks were also analyzed for intrinsic disorder, liquid–liquid phase separation propensity using FuzDrop and AutoFuz (version 2.1.0), and charge–hydropathy relationships to evaluate their biophysical properties and potential enrichment in disorder-associated behavior.

Functional enrichment analyses of these networks were also performed within STRING using Gene Ontology (GO) biological process, molecular function, and cellular component categories. Enriched terms were ranked based on false discovery rate (FDR)-adjusted significance values, which account for multiple testing and estimate the expected proportion of false-positive results among the terms considered significant, and used to identify overrepresented pathways and functions associated with PIEZO channel interactors.

### 2.5. Variant Annotation Using ClinVar

Clinically relevant variants associated with PIEZO1 and PIEZO2 were retrieved from the ClinVar database, a publicly available archive of human genetic variants and their reported relationships to disease [118].

Variants were queried by gene name and restricted to *Homo sapiens*. Only variants with annotated clinical significance were used for analysis. Clinical significance categories included pathogenic, likely pathogenic, benign, likely benign, and variants of uncertain significance (VUS) [118]. Variant information, including genomic position, protein consequence (missense, nonsense, etc.), and clinical classification, was extracted and compiled for analysis. ClinVar-derived variants were used to assess the distribution of disease-associated mutations within PIEZO1 and PIEZO2 and to make comparisons with intrinsic disorder profiles and predicted functional regions.

### 2.6. Spatial Mapping of Intrinsic Disorder, Binding Regions, and Genetic Variants

To evaluate the spatial relationships between intrinsic disorder, predicted binding regions, and clinically relevant variants, outputs from disorder prediction, ANCHOR analysis, and ClinVar were integrated and mapped onto the full-length sequences of PIEZO1 and PIEZO2.

ClinVar-derived variants were mapped to their corresponding amino acid positions within each protein sequence. Variants were categorized based on clinical significance (pathogenic, benign, VUS, etc.) and mutation type (missense, nonsense, etc.). Residue-level mappings were used to determine whether variants occurred within (i) long intrinsically disordered regions (IDRs), (ii) ANCHOR-predicted binding regions, or (iii) ordered regions. Furthermore, local disorder at ClinVar mutation sites was quantified by extracting the PONDR^®^ VSL2 score for each PIEZO1 and PIEZO2 variant residue and calculating the mean PONDR^®^ VSL2 score across a seven-residue window centered on the mutation site, corresponding to the mutation site ±3 residues.

Overlap analyses were performed by comparing residue indices across datasets. The proportion of variants located within IDRs and binding regions was calculated for each protein. To assess whether variants were preferentially enriched within specific structural categories (IDRs vs. ordered regions), contingency table analyses were conducted using Fisher’s exact tests.

All data processing, integration, and statistical analyses were performed in R (version 4.5.3) [119] using packages including dplyr (version 1.2.0) for data manipulation and tidyr (version 1.3.0) for data reshaping. Visualization of disorder profiles, ANCHOR regions, and variant distributions was performed using ggplot2 (version 4.0.2) [120]. Overlay plots were generated by aligning residue position on the X-axis with corresponding disorder scores, ANCHOR predictions, and variant positions, allowing for a visual comparison of structural and functional features along the protein sequence.

### 2.7. Structural Modeling of Mutation-Containing Peptide Regions

To evaluate the potential structural effects of select mutations, peptide fragments corresponding to variant-containing intrinsically disordered regions of PIEZO1 and PIEZO2 were modeled using PEP-FOLD4 (version 4.0) [121].

Short peptide sequences encompassing the mutation site (including flanking residues to preserve local structural context) were extracted from the full-length protein sequence. Both wild-type and mutant peptide sequences were submitted independently to PEP-FOLD4 for de novo structure prediction. PEP-FOLD4 employs a fragment-based approach combined with a coarse-grained force field and a structural alphabet representation to generate plausible peptide conformations without reliance on homologous templates [121].

For each input sequence, multiple structural models were generated, and the top-ranked models were selected based on the sOPEP (optimized potential for efficient structure prediction) energy score. Predicted structures were compared qualitatively between wild-type and mutant peptides to assess potential differences in secondary structure propensity, folding patterns, and overall conformational behavior. Because PEP-FOLD4 models isolated linear peptides, these structures were used only to compare local conformational tendencies. They do not account for the membrane environment, lipid tension, intermolecular interactions, or constraints in the full-length PIEZO proteins.

### 2.8. Evolutionary Conservation Analysis of PIEZO1 and PIEZO2

Evolutionary conservation of PIEZO1 and PIEZO2 was evaluated using ortholog sequence alignment, pairwise identity analysis, phylogenetic tree construction, and residue-level conservation scoring. Canonical human PIEZO1 and PIEZO2 protein sequences were downloaded from UniProt (uniprot.org), along with orthologous vertebrate sequences representing mammalian and non-mammalian taxa. For PIEZO1, orthologs included human (*Homo sapiens*), chimpanzee (*Pan troglodytes*), mouse (*Mus musculus*), rat (*Rattus norvegicus*), bovine/cow (*Bos taurus*), pig (*Sus scrofa*), red fox (*Vulpes vulpes*), chicken (*Gallus gallus*), Indian cobra (*Naja naja*), and zebrafish (*Danio rerio*). For PIEZO2, the same general taxonomic sampling was used, except Japanese killifish (*Oryzias latipes*) was used as the teleost representative because a zebrafish PIEZO2 ortholog was not available in the selected UniProt dataset.

Sequences were imported into Jalview (version 2.11.5.2) and aligned using Clustal Omega (version 1.2.4). Full-length alignments were used to calculate pairwise percent identity values between all orthologs. These values were exported and visualized as pairwise identity heatmaps in R. Neighbor-joining trees were generated in Jalview from the full-length alignments using the BLOSUM62 substitution matrix to visualize evolutionary relationships among the selected orthologs.

To examine conservation within predicted disordered regions, the largest high-confidence IDR or major disordered region of each protein was analyzed separately using the same Jalview alignment framework. Jalview conservation, quality, consensus, and percent identity shading were used to evaluate whether these regions showed residue-level conservation, conserved motif-like features, or species-specific insertions and deletions. IUPred disorder profiles were also inspected across aligned orthologs to determine whether disorder propensity was retained in corresponding regions even when exact amino acid identity was reduced.

Residue-level conservation was further assessed using ConSurf (github.com/BenTalLab/consurf, accessed on 3 August 2026). ConSurf analyses were performed separately for PIEZO1 and PIEZO2 using 150 selected homologs. Homolog searches were limited to a maximum redundancy of 95%, a minimum sequence identity of 35%, and an E-value cutoff of 0.0001. Homolog selection was performed using the sampling option, and multiple-sequence alignments were generated using MAFFT (version 7.526). Conservation grades were summarized across the full-length protein, non-IDR residues, all high-confidence IDRs, and the major IDR or disordered region of each protein. High-confidence IDRs were defined as regions where at least 75% of disorder predictors agreed. Mean and median ConSurf grades, along with the mean dominant amino acid percentage at each position, were used to compare the conservation of predicted disordered regions with the rest of each protein.

## 3. Results and Discussion

### 3.1. Intrinsic Disorder Profiles of PIEZO1 and PIEZO2

Intrinsic disorder analysis of PIEZO1 revealed moderate levels of disorder across predictors, with moderate variation depending on the algorithm used (Table 1). The percentage of predicted intrinsically disordered residues (PPIDRs) ranged from 14.76% (IUPred-L) to 27.13% (PONDR^®^ VSL2), while average disorder scores (ADSs) ranged from 0.188 (IUPred-L) to 0.328 (PONDR^®^ VSL2).

The consensus mean disorder profile (MDP) yielded a PPIDR of 20.31% and an ADS of 0.261, indicating that approximately one-fifth of the protein exhibits intrinsic disorder. Across prediction models, machine learning-based methods such as PONDR^®^ VSL2 and PONDR^®^ VLXT consistently identified higher levels of disorder relative to energy-based approaches like IUPred. Despite this variability, all predictors classified PIEZO1 as a moderately disordered protein. Residue-level analysis of IUPred predictors identified multiple discrete intrinsically disordered regions (IDRs), primarily localized within the C-terminal portion of PIEZO1. Seven major (>20 consecutive residues) IDRs were detected, ranging in length from 24 to 66 residues, with the longest region spanning residues 1565–1630 (66 residues). Additional regions were identified spanning residues 735–769, 1353–1406, 1423–1446, 1457–1502, 1811–1867, and 1882–1919. The comparison with curated annotations from UniProt showed strong concordance, with experimentally or computationally annotated disordered regions overlapping substantially with our predicted IDRs, particularly in the regions spanning residues 738–769, 1356–1402, 1462–1498, 1576–1630, and 1811–1921. These findings support the robustness of the disorder predictions and highlight the presence of conserved disordered segments within PIEZO1.

**Figure 2:**
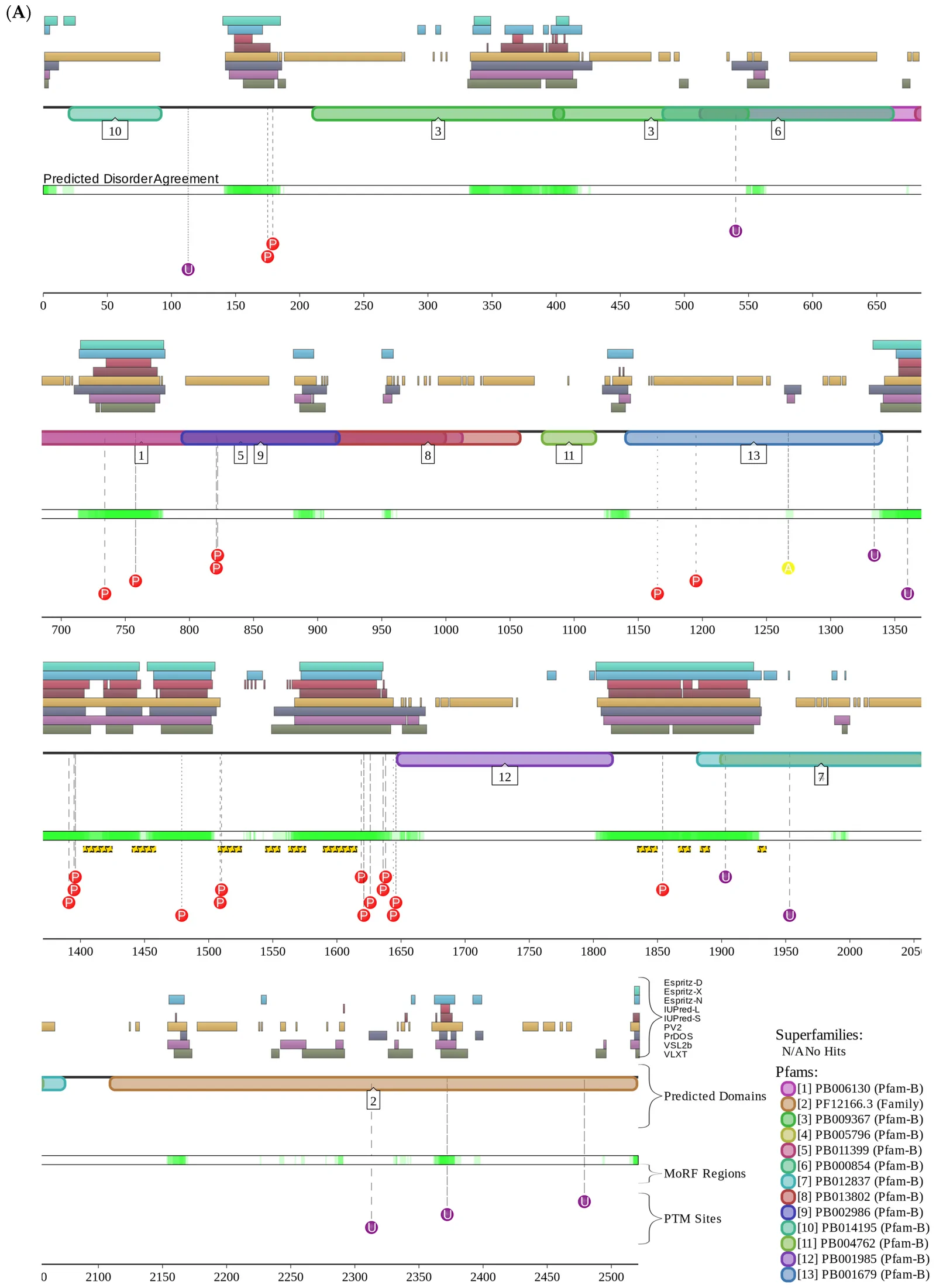

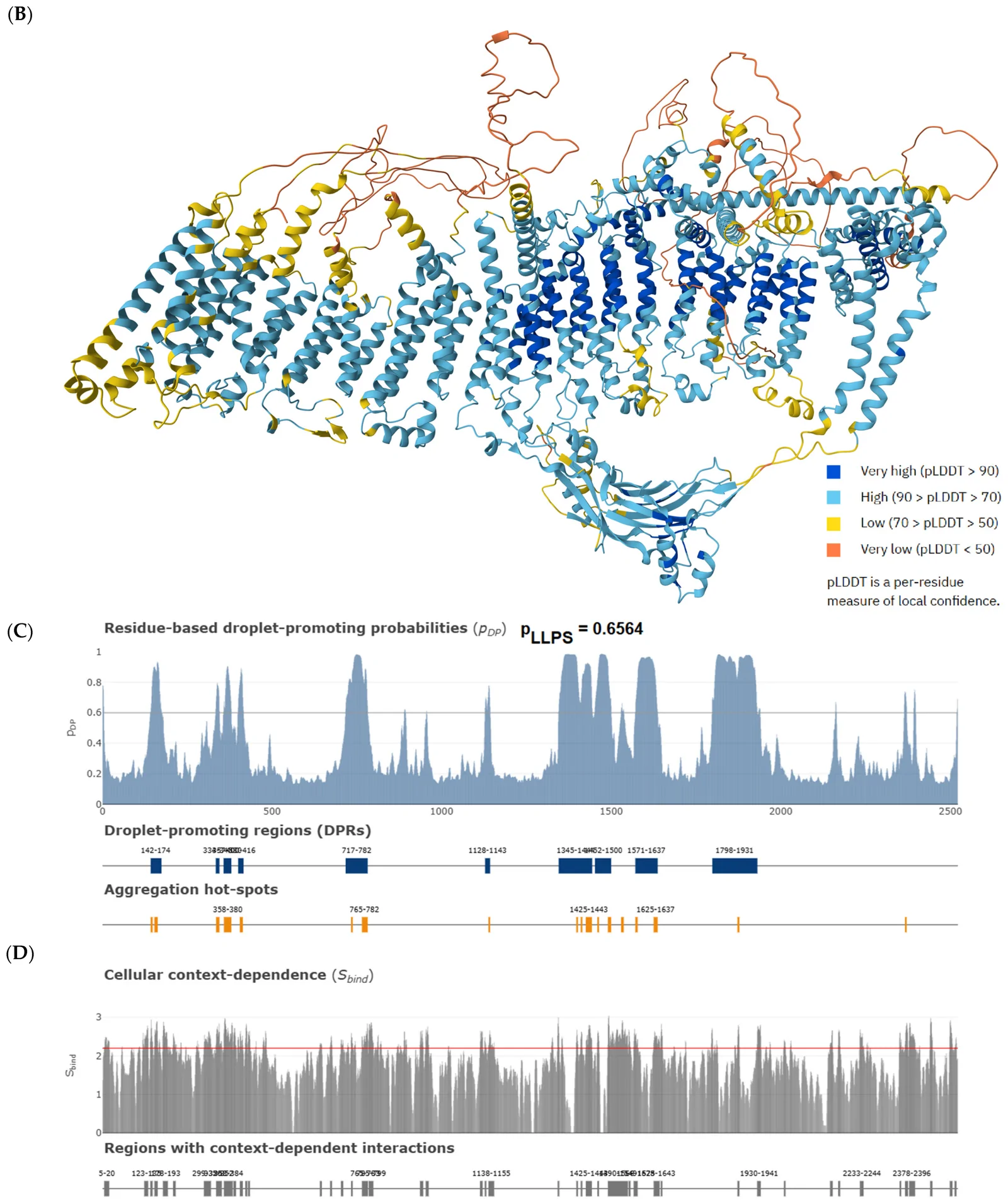
Functional disorder analysis of human PIEZO1 mechanosensitive ion channel (UniProt ID: Q92508). (**A**). Functional disorder profile generated by D^2^P^2^. The nine colored bars show locations of IDRs predicted by different tools, whereas a green-blue bar presents a consensus view of disorder across all predictors. Above this consensus bar, two lines with numbered, colored bars show the predicted locations of SCOP (Structural Classifications of Proteins) domains [123,124], identified by the SUPERFAMILY predictor [125]. Furthermore, yellow patterned bars show positions of MoRFs. Positions of PTMs are shown as differently colored circles at the bottom of the plot. (**B**). 3D structural model generated by AlphaFold (AlphaFold DB v6). Colors represent the predicted local distance difference test (p_LDDT_) scores: very high confidence (p_LDDT_ > 90, dark blue), high confidence (90 > p_LDDT_ > 70, light blue), low confidence (70 > p_LDDT_ > 50, yellow), and very low confidence (p_LDDT_ < 50, orange). (**C**). FuzDrop-based evaluation of the LLPS potential of human PIEZO1. Plot shows the sequence distribution of the residue-based droplet-promoting probabilities, p_DP_. Based on their probability of undergoing spontaneous LLPS (p_LLPS_) values and the presence of droplet-promoting regions (DPRs), proteins are classified as droplet drivers or droplet clients. Here, proteins with p_LLPS_ ≥ 0.6 are considered droplet drivers capable of spontaneous LLPS, whereas DPR-containing proteins with p_LLPS_ < 0.60 are classified as droplet clients. DPRs (shown by blue bars below the p_DP_ profiles) are identified as regions containing at least five consecutive residues with p_DP_ ≥ 0.60 [113,115]. Orange bars indicate the positions of aggregation hotspots, which are defined as droplet-promoting (p_DP_ ≥ 0.60) regions that exhibit high interaction mode divergence (S_bind_ ≥ 2.2), making them sensitive to the cellular context. (**D**). Sequence distribution of FuzDrop-predicted cellular context-dependent interaction regions (S_bind_). Regions with context-dependent interactions change protein interaction behavior and binding modes under different cellular conditions. These regions can be ordered or disordered, characterized by S_bind_ ≥ 2.25 [113,115].

Figure 2 provides further support for the presence of noticeable levels of intrinsic disorder in human PIEZO1. Figure 2A shows the functional disorder profile generated for human PIEZO1 by D^2^P^2^ platform (https://d2p2.pro/; accessed on 30 April 2026) [122].

D^2^P^2^ assembles the outputs of nine well-established computational per-residue disorder prediction models, such as PONDR^®^ VLXT [126], PONDR^®^ VSL2 [107], two IUPred versions—short (to identify short, typically 3–10 residues long, localized IDRs in proteins) and long (to predict long IDRs targeting segments of 30 or more consecutive residues) [111,127]—PrDOS [128], PV2, and three variants of ESpritz (NMR, DisProt, and X-ray trained on NMR flexibility data, DisProt database, and residues missing from Protein Data Bank (PDB) X-ray structures, respectively) [129].

Figure 2A also contains functional annotations, such as localization of functional domains, disorder-based binding regions (molecular recognition features, MoRFs, which are IDRs undergoing disorder-to-order transition upon binding to specific partner regions) identified by the ANCHOR algorithm [110,112], as well as positions of various post-translational modifications (PTMs) identified by the PhosphoSitePlus platform [130]. Figure 2A shows that PIEZO1 contains 15 high-confidence IDRs identified as regions, where at least 75% of disorder predictors agree. These IDRs (residues 149–162, 366–373, 399–401, 406–407, 735–770, 1353–1403, 1420–1421, 1423–1441, 1443, 1458–1501, 1571–1631, 1812–1867, 1870–1876, 1882–1920, and 2367–2371) are located between or within the functional domains of this protein. The presence of these regions defines the high structural flexibility of this protein and explains the presence of multiple regions with missing electron density in its experimentally determined 3D structure (see Figure 1). Therefore, based on this very conservative disorder evaluation, PIEZO1 contains at least 13.8% disordered regions. There are also 10 ANCHOR-identified MoRFs (residues 1402–1424, 1440–1458, 1507–1525, 1544–1555, 1562–1575, 1589–1615, 1834–1849, 1866–1875, 1883–1890, and 1928–1934) and multiple different PTMs. The presence of MoRFs was further supported by analyzing this protein using the MoRFpred server designed for accurate MoRF prediction [131]. This analysis revealed that human PIEZO1 is expected to contain nine MoRFs (residues 1351–1355, 1401–1408, 1415–1424, 1446–1457, 1508–1512, 1566–1568, 1805–1809, 1842–1846, and 1869–1873). The overlap and coincidence of MoRFs predicted by these two different computational tools indicate a high level of confidence in the identification of these specific disordered regions as functional binding sites. In fact, when independent algorithms with different underlying methodologies yield overlapping predictions, it strongly suggests that these segments possess a genuine, biologically relevant propensity for coupled folding and binding.

Figure 2B represents the AlphaFold-generated 3D structural model of human PIEZO1 and shows that this protein contains multiple structural elements predicted with low or very low confidence, indicating that the corresponding regions, which mostly correspond to loops and linkers, are predicted to be characterized by high structural flexibility. These low-confidence segments, often denoted by low p_LDDT_ scores, frequently correspond to IDRs that lack a fixed tertiary structure under physiological conditions. In the context of a massive, mechanically activated ion channel like PIEZO1, these flexible cytosolic and extracellular loops are not merely structural artifacts. Instead, they are highly likely to serve as critical functional linkers. They allow the rigid, blade-like transmembrane domains to undergo the large-scale conformational rearrangements required to transition between closed, open, and inactivated states during mechanical force transduction. Furthermore, these highly flexible, unstructured loops often present accessible surface areas that act as dynamic hubs for post-translational modifications and protein–protein interactions, modulating the channel’s sensitivity to membrane tension.

As seen in Figure 2C, PIEZO1 is classified as a droplet driver, since it is characterized by a high p_LLPS_ of 0.66 and contains 10 droplet-promoting regions (DPRs; residues 142–174, 334–345, 357–380, 400–416, 717–782, 1128–1143, 1345–1444, 1452–1500, 1571–1637, and 1798–1931) spread through most of the sequence. Finally, Figure 2D shows that this mechanosensitive ion channel contains multiple regions with context-dependent interactions (RCDIs), i.e., regions predicted to exhibit ordered or disordered binding modes depending on the cellular context (environment, sub-cellular localization, partners, and PTMs). All this defines PIEZO1 as a protein that is expected to utilize intrinsic disorder as an important functional means. In fact, while the core transmembrane structure of PIEZO1 consisting of 38 transmembrane α-helices is well-defined, the intracellular domains of this multi-pass transmembrane protein incorporate significant IDRs, which act as flexible functional modules, enabling the channel to adapt and respond to various cellular environments.

Similarly, intrinsic disorder analysis of PIEZO2 revealed moderate-to-high levels of disorder across predictors (Table 1). The PPIDR values ranged from 15.37% (IUPred-L) to 31.69% (PONDR^®^ VSL2), while ADS ranged from 0.196 (IUPred-L) to 0.361 (PONDR^®^ VSL2). The consensus mean disorder profile (MDP) yielded a PPIDR of 23.76% and an ADS of 0.274, indicating a higher overall disorder propensity compared to PIEZO1, but PIEZO2 was still classified as a moderately disordered protein according to our analysis.

Residue-level analysis of only the IUPred predictor identified eight major (>20 consecutive residues) IDRs in PIEZO2, ranging in length from 21 to 110 residues. Notably, a large IDR spanning residues 1716–1825 (110 residues) represented the longest continuous disordered segment identified in either protein. Additional IDRs were located at residues 453–478, 628–663, 870–901, 1495–1534, 1594–1636, 2044–2064, and 2090–2133. Comparison with UniProt annotations demonstrated strong agreement, with predicted IDRs overlapping annotated disordered regions at residues 446–478, 623–664, 862–902, 1488–1534, 1593–1636, and 2090–2135.

Significant positive linear relationships were observed between PPIDR and ADS for both PIEZO proteins. For PIEZO1, regression analysis produced a slope of 72.480 with a strong fit (R^2^ = 0.723, *p* = 0.015). Similarly, PIEZO2 showed a slope of 72.953 and an even stronger relationship (R^2^ = 0.828, *p* = 0.015). These findings indicate strong concordance between the two disorder metrics across prediction algorithms for both proteins.

**Figure 3:**
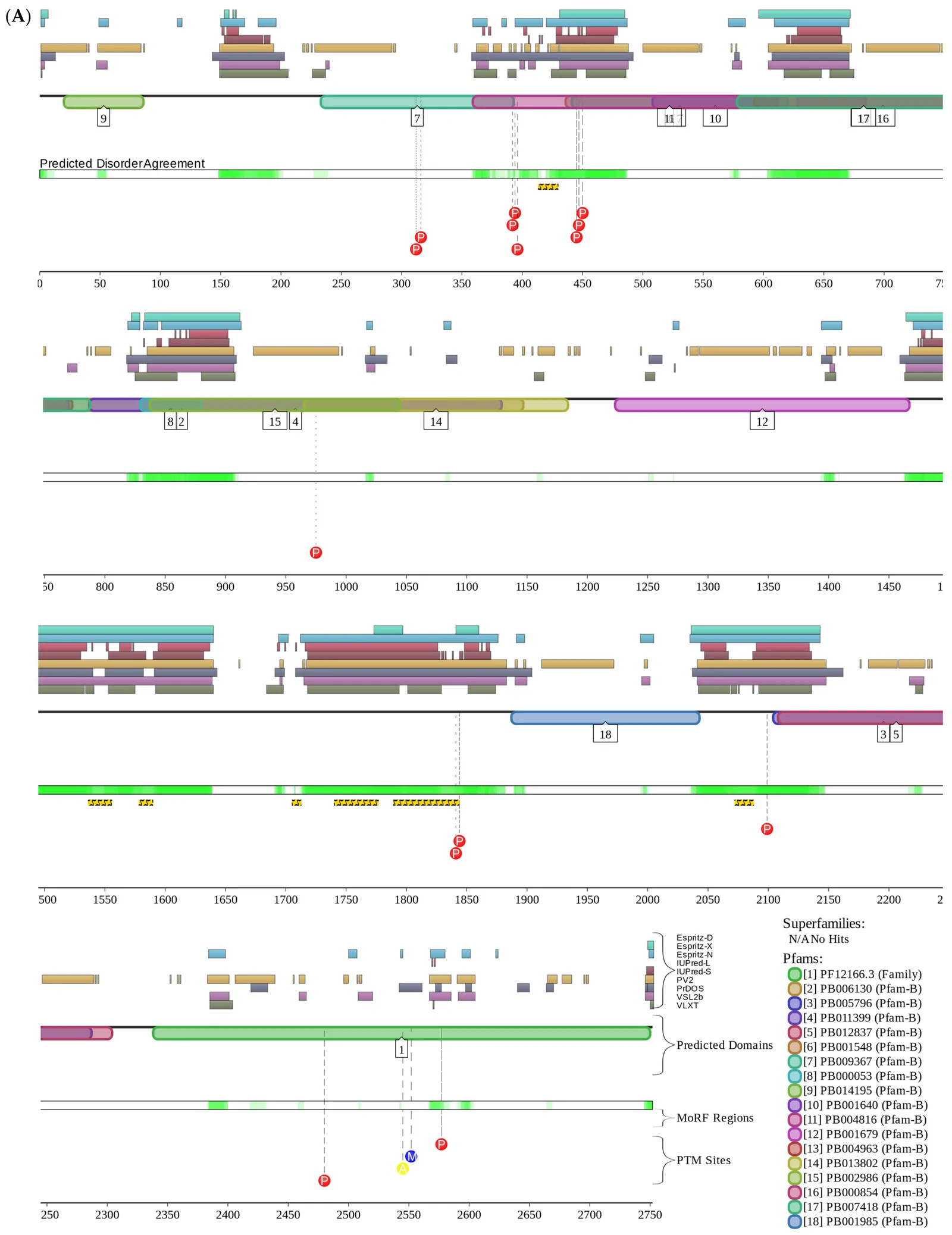

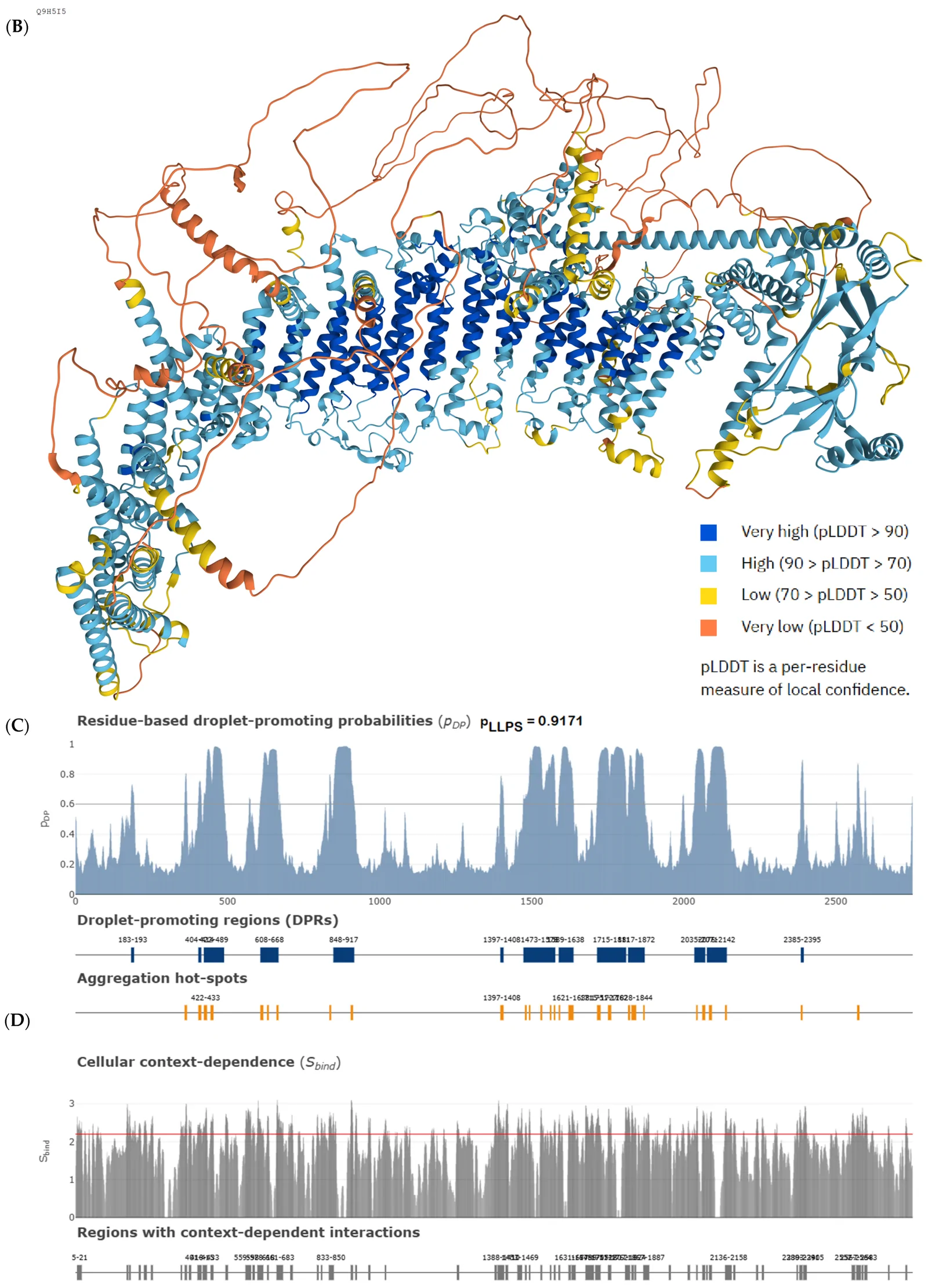
Intrinsic disorder analysis of human piezo-type mechanosensitive ion channel PIEZO2 (UniProt ID: Q9H5I5). (**A**). Functional disorder profile generated by D^2^P^2^. (**B**). 3D structural model generated by AlphaFold (AlphaFold DB v6). (**C**). FuzDrop-based evaluation of the LLPS potential of human PIEZO2. (**D**). FuzDrop-predicted cellular context-dependent interaction regions (S_bind_).

Noticeable intrinsic disorder content and remarkable disorder-based functionality of human PIEZO2 are further illustrated by Figure 3.

Figure 3 represents the functional disorder profile (Figure 3A), AlphaFold-generated structure, where the ordered part of this protein consisting of 38 transmembrane α-helices is interspersed with ribbon-like regions representing predicted intrinsically disordered segments (Figure 3B), LLPS potential (Figure 3C), and predicted regions with context-dependent interactions (i.e., segments that are expected to change protein interaction behavior and binding modes under different cellular conditions (Figure 3D)), as estimated by the FuzDrop S_bind_ score (here, S_bind_ value represents the Shannon entropy of a protein residue binding-mode distribution. It quantifies a sequence segment capacity to switch between highly diverse structural states upon interacting with different partners; the score S_bind_ ≥ 2.25 indicates a high multiplicity of binding modes, meaning the region dynamically switches between disorder and order [113,114,115]).

Figure 3A shows that human PIEZO2 contains 24 high-confidence IDRs, i.e., regions, where at least 75% of predictors used in D^2^P^2^ agree. These high-confidence IDRs (152–164, 367–367, 431–433, 438, 453–478, 628–663, 858–62, 870–872, 874–875, 877–902, 1477–1480, 1485–1485, 1488–1488, 1495–1535, 1539–1539, 1562–1573, 1594–1637, 1718–1796, 1801–1824, 1848–1859, 1866–1868, 2044–2065, and 2091–2133) cover 14.5% of the PIEZO2 sequence. Many of these IDRs are located within long intracellular domains of this protein (residues 142–221, 356–492, 615–685, 808–940, 1430–1921, 1721–1887, and 2036–2189). There are also seven MoRFs (residues 413–429, 1536–1555, 1578–1589, 1705–1712, 1740–1776, 1789–1843, and 2072–2087) (Figure 3A). Many of these ANCHOR-predicted MoRFs are supported by MoRFpred, which finds regions capable of disorder-to-order transition upon binding to specific partners at residues 176–179, 388–395, 446–458, 591–595, 630–642, 1538–1541, 1550–1552, 1580–1588, 1706–1709, 1983–1987, 2081–2083, and 2585–2589. One can also find 13 DPRs in this protein (residues 183–193, 404–414, 422–489, 608–668, 848–917, 1397–1408, 1473–1578, 1589–1638, 1715–1811, 1817–1872, 2035–2071, 2078–2142, and 2385–2395) (Figure 3C). Figure 3C also shows that PIEZO2 is characterized by a very high p_LLPS_ of 0.92, suggesting that this protein is capable of spontaneous LLPS, acting as a strong droplet driver. Similar to PIEZO1, PIEZO2 contains a very large number of RCDIs (see Figure 3D).

In line with these considerations, a recent study provided compelling evidence of the functional importance of the mouse PIEZO2 intracellular intrinsically disordered domains [15]. In addition to numerous short loops and linkers, this protein has seven large intracellular domains (residues 142–221, 356–492, 615–689, 812–957, 1448–1680, 1705–1991, and 2106–2259) that together constitute ~25% of its sequence and account for the majority of the size and sequence differences between PIEZO2 and PIEZO1 [15]. These long IDRs of PIEZO2 represent intracellular domains, which were defined as intrinsically disordered domains 1–7 (IDR1–7) [15]. Furthermore, it was established that these intracellular IDRs of PIEZO2 are responsible for controlling some key functional features of this protein [15,132]. In fact, experimental analysis showed that while all individual IDR deletion mutants remained functional, removing specific intracellular domains altered distinct functional features of this mechanosensitive ion channel [15]. For example, the deletion of IDR2 (the longest intracellular domain of PIEZO2, which connects the clasp domain with transmembrane helical unit 8), IDR3 (links the clasp domain to the latch domain), and IDR5 (linker between the transmembrane helices 12 and 13) altered membrane indentation-evoked PIEZO2 currents, where IDR2 fine-tuned the velocity sensitivity of PIEZO2, IDR3 was required for lateral plug function, and IDR5 was necessary for PIEZO2 activation by cytoskeleton-transmitted forces [15]. The lateral plug is a short peptide representing a specialized domain on the intracellular side of the channel that acts as a physical gate blocking the channel’s lateral ion-conducting pathways until mechanical action forces it open. It helps in regulating ion flow through the channel’s lateral portals. Removing this plug increases ion conductance by allowing ions to pass through these side openings [17], highlighting the important roles of IDR3 in normal PIEZO2 gating. The deletion of IDR4 increased the stretch sensitivity of PIEZO2 but did not affect its poking sensitivity [15]. It was hypothesized that the interplay between the intrinsically disordered intracellular domains of PIEZO2 [15] and the intrinsically disordered ectodomains of transmembrane syndecans [133] creates a dynamic, adaptable interface that fine-tunes mechanosensitivity and facilitates the transmission of mechanical signals or “force-from-filament” cues from the extracellular matrix directly to the PIEZO2 channels [134].

Importantly, both PIEZO1 and PIEZO2 proteins serve as a perfect illustration of the proteoform concept. In fact, according to this concept, a single gene does not simply produce one protein but generates an ensemble of unique molecular entities due to genetic variations, alternative RNA splicing, PTMs, intrinsic disorder, and function [38,42,43]. An example of the alternative spicing-driven regulation of these proteins is given by their plug domains. Both proteins undergo this process, which is essential for their unique “plug-and-latch” system. This system physically controls the opening of the side portals that conduct ions. Because splicing determines the presence of the lateral plug gate, which regulates ion permeation through the lateral portals, it directly changes how the channel behaves. For instance, variants without the standard plug gate show increased electrical conductance and higher sensitivity to mechanical force. Furthermore, disrupting the latch mechanism can cause the channel to enter alternative sub-conductance states [17]. Figure 2 and Figure 3 show that both proteins contain multiple different PTMs, which further increases their structural and functional diversity.

Taken together, these observations indicated that IDRs play crucial roles in the functionality of this polymodal mechanosensor that detects various mechanical stimuli through separate force transmission pathways. Because IDRs lack a fixed structure, they can act like flexible tethers or “springs” that connect different domains of the protein. Their flexibility allows the sensor to integrate multiple signals by using different “arms” to “feel” shear stress, membrane tension, or poke-induced pressure. These IDRs function as critical regulators in protein mechanics and signaling by acting as dynamic, force-sensitive switches that alter conformational pathways based on mechanical tension. By changing their shape in response to pulling, IDRs allow a single protein to toggle between different functional states or signaling outcomes. Finally, by adjusting their tension, IDRs can fine-tune how much force is required to open the pore and control channel sensitivity.

### 3.2. Liquid–Liquid Phase Separation Propensity

PIEZO1 displayed a high LLPS probability (p_LLPS_ = 0.66, exceeding the 0.6 threshold), with several droplet-promoting regions in the N-terminal and central portions of the protein (see Figure 2C). This suggests that PIEZO1 has sequence features associated with the spontaneous formation of droplets in the cytoplasm, which may create high-concentration environments. PIEZO2 had a noticeably higher LLPS probability (p_LLPS_ = 0.92), with many long DPRs distributed throughout the protein (see Figure 3C). This stronger LLPS propensity is consistent with the higher disorder levels and longer intrinsically disordered segments identified in PIEZO2; however, experimental validation is required.

FuzDrop analysis revealed that PIEZO1 contains 10 DPRs that cover ~20.5% of its sequence (516 of 2521 residues). Taken together, these DPRs are characterized by high fractions of charged and polar residues (such as E (14.3%), D (5.8%), R (10.3%), S (7.2%), and Q (7.4%)), and contain high fractions of helix-breaker P (9.7%), G (9.1%), and A (10.3%), which prevent folding of these regions and promote their high structural flexibility, maximizing conformational entropy. Some of these regions contain massive clusters of negatively charged residues (such as EEEEEEED and EEEEE) balanced by patches of positively charged R and K. These opposite charges create weak, shifting salt bridges across different chains. DPRs also contain QQQQQ repeats and P/G-rich patches that create further foundation for the multivalent, weak interactions necessary to form liquid condensates. Furthermore, the aromatic rings of Y, F, and W scattered throughout the sequences facilitate the π-π interactions, whereas the abundant R residues interact dynamically with aromatic residues forming the cation–π contacts required to drive phase separation without needing an external scaffold. On the contrary, hydrophobic amino acids, such as I (1%), C (0.4%), and M (0.6%), which normally drive the formation of a tightly packed hydrophobic core, are almost entirely absent. As a result, these sequences remain fully exposed to the solvent, allowing them to condense into a macroscopic fluid droplet rather than precipitating or folding tightly. Because these sequences sample an endless continuum of disordered binding modes, the free energy barrier to transition into a liquid droplet is incredibly low. The chains balance enough weak attractive forces to aggregate with enough structural disorder to keep the resulting assemblage completely fluid.

Similarly, 13 DPRs that cover 23.8% of the human PIEZO2 sequence lack rigid order-promoting structural domains and are heavily biased toward the amino acids that favor intrinsic disorder (E (18.5%), S (11.5%), K (8.7%), D (7.5%), R (7.0%), G (6.7%), P (5.0%), and Q (5.0%)). They also contain repeating motifs and charge blocks (such as clustered D/E and R/K residues) that provide multiple, alternative, non-specific contact sites, and the segregation of charged patches allows multiple protein chains to dynamically align and self-associate through weak, alternating salt bridges. Multiple arginine residues contribute to multivalency as well due to the ability of this residue to participate in electrostatic interactions, hydrogen bonding, and cation–π interactions. Aromatic residues dispersed throughout some DPRs drive liquid–liquid phase separation (LLPS) by forming multivalent π-π stacking and cation–π contacts with charged residues like arginine. These dispersed residues act as sticky interaction sites (“stickers”) that stabilize biomolecular condensates [75,135].

We also analyzed the LLPS predispositions of human PIEZO1 and PIEZO2 proteins using the Molecular Condensation and Phase Separation Predictor, MolPhase, an advanced algorithm that leverages diverse physicochemical features and extensive experimental data to deliver accurate protein phase separation predictions [116]. MolPhase analysis revealed that human PIEZO1 and PIEZO2 proteins exhibit prediction scores of 0.990 and 0.991, respectively. These near-maximum scores indicate an exceptionally high computational propensity for these proteins to undergo LLPS, strongly suggesting that both query proteins possess the specific “molecular grammar” (such as precise configurations of stickers and spacers, electrostatic–π interactions, or disordered domains) required to drive cellular condensate assembly. Since both FuzDrop and MolPhase indicate that human PIEZO1 and PIEZO2 have high LLPS potential, this convergent evidence from two distinct architectural philosophies strongly reinforces the hypothesis that these mechanosensitive channels might function within macromolecular condensates. While FuzDrop focuses heavily on long-range conformational entropy and disorder-driven spontaneous phase separation, MolPhase validates this by scoring the proteins based on specific physicochemical features like amino acid composition, charge patterning, and sticker–spacer architectures. The near-maximum scores from MolPhase (0.990 for PIEZO1 and 0.991 for PIEZO2) and high p_LLPS_ scores from FuzDrop (0.66 and 0.92, respectively) elevate this from a tentative observation to a highly robust in silico prediction. This suggests that the large intracellular loops or terminal domains of PIEZO proteins likely serve as hubs for multivalent interactions, potentially organizing specialized signaling microdomains at the plasma membrane or mechanosensitive hubs within the cell.

The presence of droplet-promoting regions (DPRs) in PIEZO1 and PIEZO2 proteins was further validated by utilizing the ParSe 2.0 (Partition Sequence) algorithm, which accurately predicts phase-separating intrinsically disordered regions (PS-IDRs) within the amino acid sequences of query proteins [136]. This analysis revealed that human PIEZO1 is predicted to have two PS-IDRs (residues 1376–1401 and 1597–1620) embedded within two DPRs predicted by FuzDrop (residues 1345–1444 and 1571–1637). Two PS-IDRs were found in human PIEZO2 as well (residues 1839–1865 and 2104–2126), which are embedded within the FuzDrop-predicted DPRs (residues 1817–1872 and 2078–2142). This nested architecture strongly supports the ability of the identified regions to undergo LLPS. Once again, because the regions potentially facilitating LLPS were identified by two independent algorithms, the reliability and predictive accuracy of these findings are significantly enhanced. When multiple computational tools with distinct underlying biophysical principles, such as ParSe 2.0 (which focuses on sequence partitioning and intrinsic disorder) and FuzDrop (which evaluates droplet-promoting probabilities), converge on the exact same regions, the likelihood of false positives is greatly reduced. This cross-validation provides a robust theoretical justification for moving forward with in vitro and in vivo experimental testing to confirm the actual LLPS behavior of PIEZO1 and PIEZO2.

In line with these considerations, spatial clustering and functional cooperativity were suggested to play important roles in the regulation of the activity of PIEZO ion channels [137]. Cooperativity is driven by how closely channels cluster and how well they work together. Evidence shows that PIEZO1 does indeed group together in vivo; it is spatially enriched at the retracting edges of healing skin cells [138] and forms submicrometer clusters in red blood cells, specifically in high-tension areas [139]. Similar “punctate” or dotted patterns have also been seen in studies on the effects of cholesterol on PIEZO1 activity [30]. Furthermore, using super-resolution microscopy, it was shown that the protein clusters formed by PIEZO1 in the cell membrane range in size from isolated single channels to massive assemblies over 10,000 nm^2^, and that these PIEZO1 clusters are trapped within a dynamic network of cholesterol domains, which act as boundaries that restrict their two-dimensional movement across the cell surface [30]. Although the movements of the PIEZO clusters within the membrane are controlled by membrane composition, the FuzDrop results suggest that LLPS may contribute to the formation of these clusters. Therefore, such membrane-embedded clusters of PIEZO channels may represent a possible example of 2D phase separation [45].

Our analysis also revealed that both human PIEZO channels contained multiple RCDIs (see Figure 2D and Figure 3D), showing that phase behavior and clustering may be influenced by binding partners, concentration, or the cellular environment. Of 36 such regions found in PIEZO1, 7 regions with context-dependent interactions (residues 1410–1415, 1425–1443, 1490–1549, 1551–1557, 1566–1578, 1872–1878, and 1930–1941) overlap with MoRFs. Similarly, 8 of the 67 PIEZO2 RCDIs (residues 418–433, 1573–1579, 1709–1727, 1807–1824, 1828–1836, 1838–1844, 2074–2079, and 2083–2093) overlap with or are embedded into MoRFs as well. The enrichment of MoRFs within RCDIs underscores a sophisticated regulatory mechanism for both PIEZO1 and PIEZO2. These overlapping domains function as localized structural switches. By transitioning from intrinsically disordered states to structured conformations upon binding, these regions allow the mechanosensitive channels to dynamically sample different conformational landscapes. This dual-nature architecture suggests that the mechanical gating threshold of PIEZO channels is not static but rather tuned by localized structural transitions driven by environmental cues or specific intracellular binding partners. Such properties are characteristic of regulatory IDPs that transition between dispersed and condensed states [8,44].

Finally, Figure 2C and Figure 3C also show that both mechanosensitive ion channels contain numerous short aggregation hotspots, which are capable of sampling both ordered and disordered binding modes and may drive the conversion of the liquid-like condensed state into a solid-like amyloid state, thereby potentially linking PIEZO1 and PIEZO2 to abnormal aggregation-related pathologies, similar to other neurodegenerative-associated proteins such as tau, FUS, TDP-43, or α-synuclein.

This phase transition from dynamic, reversible liquid droplets to irreversible, highly structured amyloid fibrils represents a critical pathogenic tipping point. In healthy physiological states, the intrinsic disorder within specific domains of PIEZO1 and PIEZO2 likely facilitates transient, functional macromolecular interactions necessary for mechanical signal transduction and membrane remodeling. However, the high density of hydrophobic and aromatic residues within these identified hotspots drastically lowers the kinetic barrier for aberrant self-association. Under conditions of cellular stress, altered membrane tension, or aging-related proteostatic decline, these local regions are expected to shift from sampling flexible, disordered conformations to locking into rigid, cross-β-sheet architectures. The expected structural consequence of this conversion is two-fold. First, the formation of solid-like aggregates within or adjacent to the mechanosensitive apparatus is poised to physically restrict the gating kinetics of PIEZO channels. This loss of conformational flexibility could lead to either chronic mechanical desensitization or sustained, pathological calcium influx. Second, like the prionic behavior observed in tau, α-synuclein, and other neurodegeneration-related aggregating proteins, these PIEZO amyloid seeds may template the misfolding of neighboring proteins, propagating cellular dysfunction across the lipid bilayer. Consequently, these findings have the potential to expand our understanding of mechanobiology, suggesting that PIEZO1 and PIEZO2 are not merely passive sensors of physical force but can act as active participants in the degenerative cascade of proteinopathies. One should keep in mind that although the described scenario is intriguing, there is no current scientific evidence indicating that these proteins can undergo self-aggregation or fibril formation. Therefore, this hypothesis requires rigorous experimental validation to confirm whether PIEZO1 and PIEZO2 actually undergo liquid-to-solid phase transitions in vivo or in vitro.

These findings suggest that PIEZO channels may not only contain flexible regulatory regions but may also participate in transient higher-order assemblies involved in channel clustering, trafficking, or localized mechanotransduction signaling. It is also possible that the stronger LLPS propensity of PIEZO2 may be particularly relevant to its sensory functions.

### 3.3. Protein–Protein Interaction Networks and Gene Ontology of PIEZO Channels

**Figure 4:**
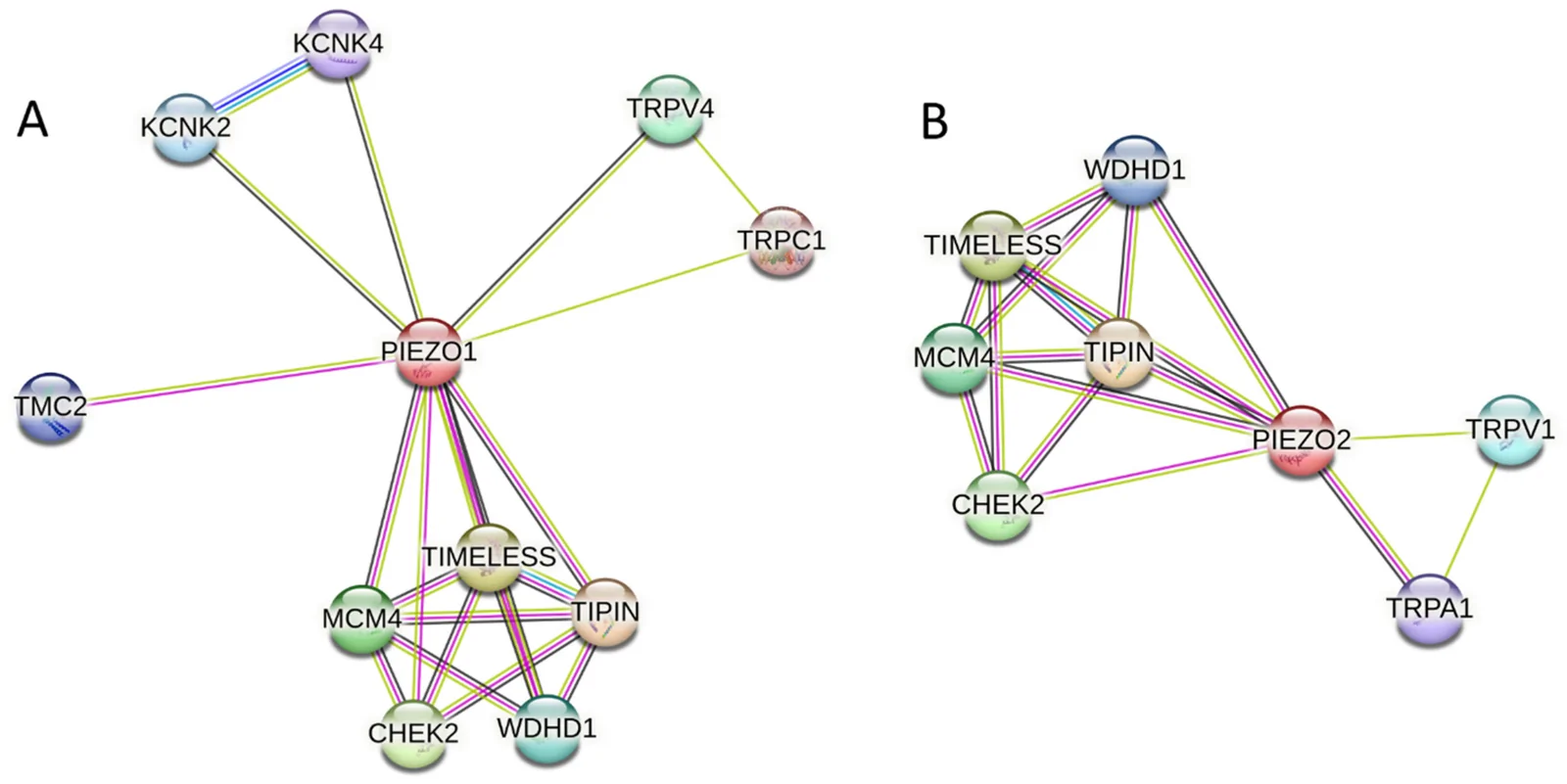
STRING protein–protein interaction network for human PIEZO1 and PIEZO2. (**A**) PIEZO1 is shown as the central node with predicted and known functional associations with mechanosensitive and ion channel proteins including TRPV4, TRPC1, TMC2, KCNK2, and KCNK4, as well as replication and cell cycle-associated proteins including TIMELESS, TIPIN, MCM4, CHEK2, and WDHD1. (**B**) PIEZO2 is shown as the central node with predicted and known functional associations with sensory ion channel proteins like TRPV1 and TRPA1, along with replication and genome maintenance proteins like TIMELESS, TIPIN, MCM4, CHEK2, and WDHD1.

Protein–protein interaction (PPI) network analysis using the STRING database with a minimum interaction score of 0.7 (high confidence) revealed that both PIEZO1 and PIEZO2 are embedded within significantly enriched and connected interaction networks (Figure 4).

The PIEZO1 network consisted of 11 nodes and 21 edges, exceeding the expected number of edges (10), with a significant enrichment *p*-value of 0.00227 (see Figure 4A). Similarly, the PIEZO2 network contained 8 nodes and 17 edges, also exceeding the expected number of edges (7), with a PPI enrichment *p*-value of 0.00141 (see Figure 4B). These results indicate that both networks had greater connectivity than expected by chance, supporting the biological relevance of the identified interactions. The network topology metrics were similar between the two proteins. PIEZO1 had an average node degree of 3.82 and a clustering coefficient of 0.904, while PIEZO2 showed a slightly higher average node degree (4.25) and a similarly high clustering coefficient (0.897), indicating tightly interconnected interaction networks in both cases.

Functionally, both proteins were associated with ion channel activity and sensory signaling pathways. PIEZO1 interactions included mechanosensitive and cation channel-associated proteins such as TRPV4, TRPC1, and TMC2, as well as potassium channel components (KCNK2 and KCNK4), consistent with its role in mechanotransduction. In contrast, PIEZO2 interactions included sensory-associated channels such as TRPV1 and TRPA1, reflecting its specialized role in sensory perception.

Analysis of functional enrichment also supported these roles. PIEZO1 showed strong enrichment in mechanosensitive ion channel activity (FDR = 9.99 × 10^−7^) and response to mechanical stimulus (FDR = 5.6 × 10^−4^). PIEZO2 showed enrichment in temperature-gated ion channel activity (FDR = 0.0071) and the detection of chemical stimuli involved in sensory perception (FDR = 0.0038). Both proteins also showed enrichment in the detection of abiotic stimuli and ion transport-related functions.

Notably, both networks contained a secondary cluster of proteins associated with DNA replication and cell cycle regulation, including MCM4, CHEK2, WDHD1, TIMELESS, and TIPIN. Enrichment in processes such as replication fork arrest and DNA-templated DNA replication (FDR ≈ 0.002–0.007) was observed in both networks. These associations raise the possibility of previously underappreciated connections between mechanosensory signaling and cell cycle regulation in PIEZO1 and PIEZO2.

**Figure 5:**
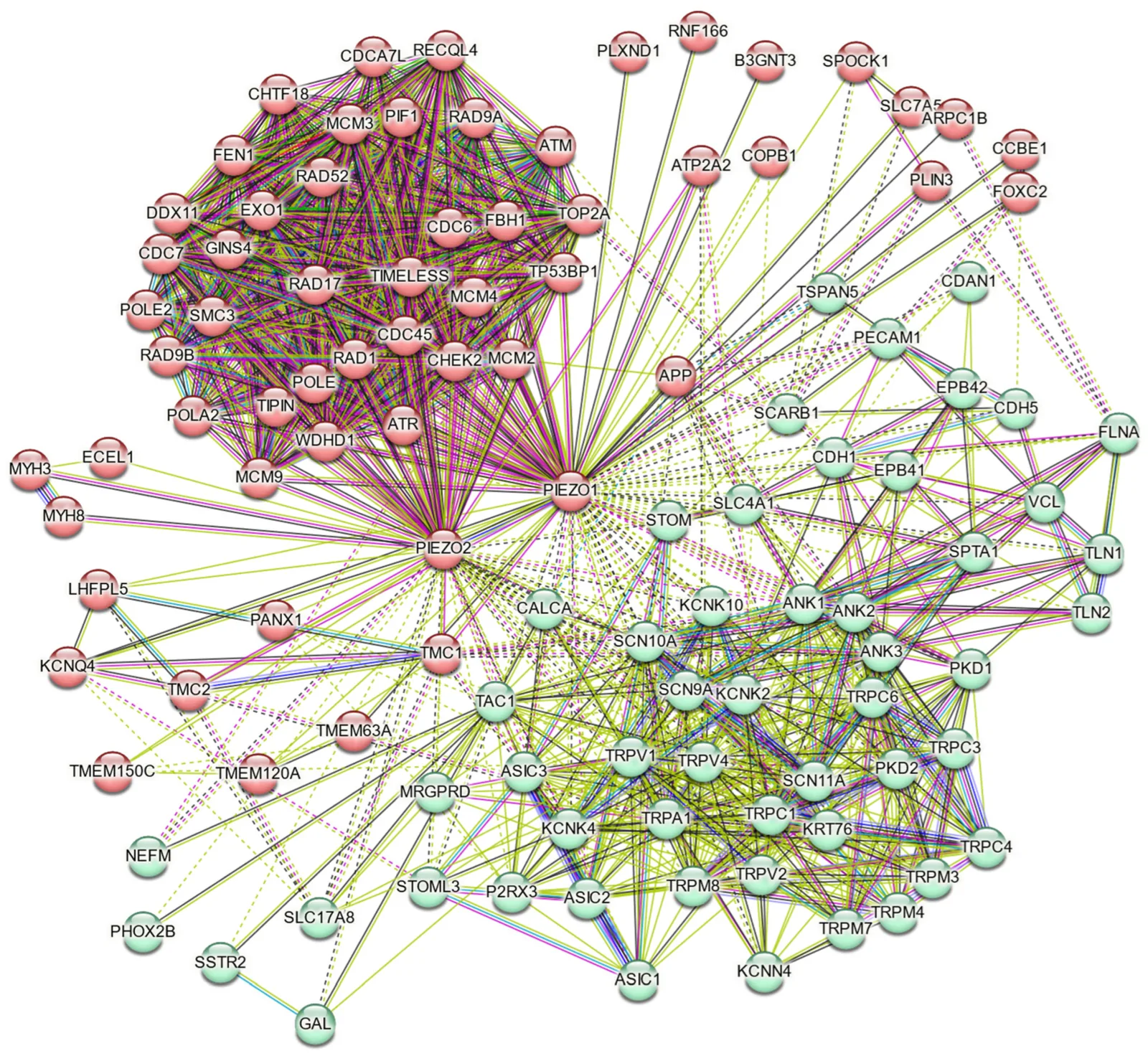
Joint protein–protein interaction (PPI) network of human PIEZO1 and PIEZO2 generated using STRING at medium confidence (interaction score = 0.4). PIEZO1 and PIEZO2 occupy central positions within a highly interconnected network containing mechanosensory ion channels, cytoskeletal adaptors, and signaling-associated proteins, including TRP channels, ankyrins, spectrins, and adhesion-related proteins. Node colors indicate STRING-derived clusters, and colored edges represent predicted or known functional associations. Interactive version of this network is available at the following permalink: https://version-12-0.string-db.org/cgi/network?networkId=b0bixhvGhAKz; accessed on 1 May 2026.

Next, when PIEZO1 and PIEZO2 were analyzed together in a combined STRING interactome (medium confidence, interaction score = 0.4), the resulting network was substantially larger and more interconnected than the individual networks (Figure 5).

The joint network had 111 nodes and 988 edges, far exceeding the 230 edges expected by chance, with a highly significant PPI enrichment value (*p* < 1.0 × 10^−16^). Network topology metrics also showed strong organization, with an average node degree of 17.8 and a clustering coefficient of 0.756. Members of this network are involved in a very broad set of cellular functions. This is reflected in their statistically significant functional enrichment in the following STRING-defined functional categories: 271 Gene Ontology (GO) terms related to biological processes, 67 molecular function GO terms, 62 cellular component GO terms, 31 local network clusters (derived in STRING using hierarchical clustering algorithms), 4 pathways from the KEGG (Kyoto Encyclopedia of Genes and Genomes) bioinformatics resource [140,141,142,143], 47 pathways from the Reactome Knowledgebase [144,145,146], 38 disease–gene associations from the DISEASES database [147], 39 tissue expressions from an integrated web resource TISSUES [148], 73 subcellular compartments from a web-based resource COMPARTMENTS [149], and 290 human phenotypes from the Monarch Initiative [150,151].

Within the combined network, PIEZO1 and PIEZO2 were in central positions linking several major protein clusters. A prominent mechanosensory cluster had multiple transient receptor potential (TRP) channels, including TRPV1, TRPV2, TRPV4, TRPA1, TRPC family members, and TRPM family members, with potassium channels such as KCNK2, KCNK4, and KCNK10. Additional highly connected subnetworks were cytoskeletal and adhesion-associated proteins such as ankyrins (ANK1–3), spectrins (SPTA1, SPTA1), talins (TLN1/2), vinculin (VCL), filamin A (FLNA), and cadherin-associated proteins. This suggests that there is extensive structural coupling between PIEZO channels and membrane scaffolding systems.

Functional enrichment analysis of the combined interactome further supported these relationships. Overrepresented Gene Ontology terms included cation channel activity, ion channel activity, calcium ion transmembrane transporter activity, and calcium channel activity, while cellular component categories were enriched in membrane and channel complex localization. In addition, a distinct secondary cluster composed of DNA replication and genome maintenance proteins, including MCM family proteins, RAD proteins, CHEK2, ATR, and TIMELESS, was retained in the merged network.

The presence of intrinsic disorder observed in both proteins may contribute to their interaction capacity, as IDRs are known to facilitate flexible and transient protein–protein interactions [34,93]. Collectively, these findings show that PIEZO1 and PIEZO2 are involved in a broad shared interaction landscape with mechanotransduction, calcium signaling, cytoskeletal organization, and potential links to cell cycle or DNA damage response pathways.

**Figure 6:**
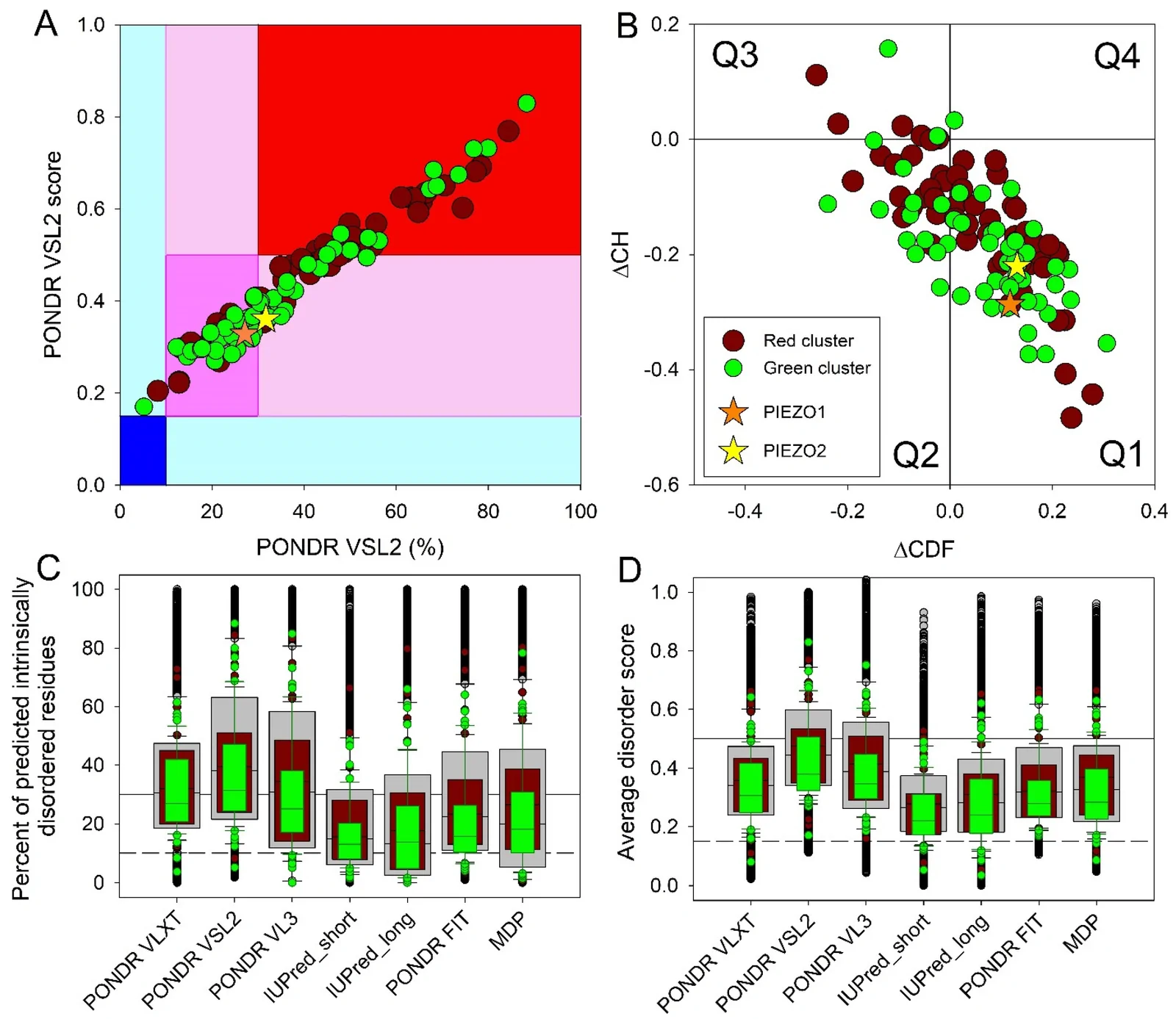
Global disorder analysis of human PIEZO1–PIEZO2-interacting proteins from the red and green clusters of the STRING-generated PPI network. (**A**). The PONDR^®^ VSL2 score (ADS) vs. PONDR^®^ VSL2 (%) (PPIDR) plot. Here, each point corresponds to a query protein, coordinates of which are evaluated from the corresponding PONDR^®^ VSL2 data as its ADS and PPIDR values. Color blocks are used to visualize proteins based on the accepted classification, with red, pink/light pink, and blue/light blue regions containing highly disordered, moderately disordered, and ordered proteins, respectively (see text for details). Dark blue or pink areas correspond to the regions where PPIDR agrees with ADS, whereas areas in which only one of these criteria applies are shown by light blue or light pink. Data for the MICAL interactome are shown by gray circles, whereas the positions of individual MICALs are shown by differently colored symbols. (**B**). Charge–Hydropathy and Cumulative Distribution Function (CH-CDF) analysis of query proteins. The ΔCH-ΔCDF plot is a two-dimensional representation that integrates the CH plot, which correlates a protein’s net charge with its hydrophobicity, and the CDF, which correlates disorder scores with their cumulative frequency. In the resulting ΔCH-ΔCDF plot, the Y-axis (ΔCH) represents the protein’s distance from the CH boundary, while the X-axis (ΔCDF) represents the deviation of a protein’s disorder frequency from the CDF boundary. Proteins are then stratified into four quadrants: Quadrant 1 (bottom right) indicates proteins likely to be structured; Quadrant 2 (bottom left) includes proteins that may be in a molten globule state or lack a unique 3D structure; Quadrant 3 (top left) consists of proteins predicted to be highly disordered; Quadrant 4 (top right) captures proteins that present a mixed prediction of being disordered according to CH but ordered according to CDF. (**C**). Nested box plot showing distributions of proteins based on their PPIDR values evaluated by seven per-residue disorder predictors (PONDR^®^ VLXT, PONDR^®^ VSL2, PONDR^®^ VL3, IUPRed_Short, IU-PRed_Long, PONDR^®^ FIT, and MDP). Horizontal lines show 10% (dotted lines) and 30% PPIDR thresholds. (**D**). Nested box plot showing distributions of proteins based on their ADS values evaluated by seven per-residue disorder predictors. The horizontal lines represent 0.15 (dotted lines) and 0.5 ADS thresholds, respectively. Corresponding data for human proteome are shown for comparison (gray boxes).

Figure 6 provides the disorder-centric view of the proteins interacting with human PIEZO1 and PIEZO2. Figure 6A plots the PONDR^®^ VSL2 score (ADS) against the percentage of disordered residues (PPIDR) for query proteins. PPIDR values classify proteins as mostly ordered (<10%), moderately disordered (10–30%), or highly disordered (>30%) [152,153], while ADS values offer a complementary classification, with ADS < 0.15, 0.15 ≤ ADS < 0.5, and ADS ≥ 0.5 corresponding to highly ordered, moderately disordered/flexible, or highly disordered proteins [153].

Because PPIDR and ADS are complementary rather than identical, they can produce differing results, making their combination a powerful way to refine protein disorder classification. Specifically, this approach identifies two distinct “plus” categories for edge cases: proteins with low PPIDR (<10%) but high ADS (≥0.15) are classified as “mostly ordered plus,” while those with moderate ADS (0.15 ≤ ADS < 0.5) but high PPIDR (>30%) fall into the “moderately disordered plus” category. These “plus” classifications are particularly valuable for distinguishing proteins with a few highly disordered regions from those with many slightly unstable regions. In corresponding PONDR^®^ VSL2 score (ADS) vs. PONDR^®^ VSL2 (%) (PPIDR) plot, this is reflected by blocks with different color intensities, where darker colors correspond to the regions where PPIDR agrees with ADS (i.e., contain mostly ordered, moderately disordered, or highly disordered proteins), whereas lighter-colored blocks contain “mostly ordered plus” and “moderately disordered plus” proteins.

Based on these metrics, the vast majority of human proteins from the joint PIEZO1–PIEZO2 interactome are classified as moderately disordered or highly disordered, as no proteins fall into the mostly ordered category and just two proteins belong to “mostly ordered plus” category (see Figure 6A). For comparison, the distribution of human proteins across the mostly ordered, mostly ordered plus, moderately disordered, moderately disordered plus, and highly disordered categories is 0.4%, 5.1%, 33.7%, 21.0%, and 39.8%, respectively [154]. The red PIEZO interactor cluster shows a similar distribution (0.00%, 1.72%, 31.04%, 32.76%, and 34.48%), whereas the green cluster is somewhat more ordered (0.00%, 1.89%, 43.40%, 28.30%, and 26.41%, respectively).

Figure 6B represents the results of global disorder analysis of human proteins interacting with PIEZO1 and PIEZO2 in the form of the ΔCH-ΔCDF plot that can also be used for the classification of proteins as mostly ordered, molten globule-like or hybrid, or highly disordered based on their positions within the resulting CH-CDF phase space [155,156,157,158]. This analysis provided further support for the idea that the proteins from the joint PIEZO1–PIEZO2 interactome include noticeable levels of disorder (63.79%, 27.59, 8.26%, and 0.00% of proteins from the red cluster and 69.81%, 24.53%, 3.77%, and 1.89% proteins from the green cluster can be found in quadrants Q1, Q2, Q3, and Q4, respectively). These disorder levels (especially in the green cluster proteins) are a bit lower than those found in the human proteome, which contains 59.1%, 25.5%, 12.3%, and 3.1% proteins in quadrants Q1, Q2, Q3, and Q4, respectively [154].

Figure 6C,D shows that the PIEZO1–PIEZO2 interactors are characterized by various levels of intrinsic disorder, being, in general, not very different from those observed in the entire human proteome. In fact, based on the seven per-residue disorder predictors used in this study (PONDR^®^ VLXT, PONDR^®^ VSL2, PONDR^®^ VL3, IUPRed_Short, IUPRed_Long, PONDR^®^ FIT, and MDP), interactors in the red cluster are characterized by the mean PPIDR values of 32.83 ± 15.15%, 40.22 ± 18.72%, 34.46 ± 20.55%, 18.64 ± 13.62%, 20.64 ± 17.89%, 26.62 ± 17.31%, and 27.35 ± 19.22 in comparison with 34.1 ± 20.3%, 43.6 ± 26.2%, 36.6 ± 29.0%, 30.4 ± 24.0%, 21.2 ± 19.3%, 22.8 ± 24.8%, and 28.3 ± 26.3% evaluated for the entire proteome. The mean corresponding PPIDR values for interactors in the green cluster are 31.04 ± 13.72, 37.00 ± 18.69, 29.94 ± 18.63, 16.32 ± 11.65, 18.02 ± 16.64, 22.29 ± 16.32, and 22.69 ± 18.11. The mean ADS values of the proteins from the red cluster of the joint PIEZO1–PIEZO2 interactome are 0.35 ± 0.12, 0.46 ± 0.13, 0.40 ± 0.13, 0.26 ± 0.10, 0.29 ± 0.14. 0.33 ± 0.12, and 0.34 ± 0.13, whereas interactors in the green cluster are characterized by the corresponding mean ADS values of 0.33 ± 0.12, 0.43 ± 0.14, 0.37 ± 0.13, 0.25 ± 0.10, 0.27 ± 0.14, 0.31 ± 0.12, and 0.32 ± 0.12. For comparison, the members of the human proteome are characterized by the corresponding mean ADS values of 0.36 ± 0.17, 0.48 ± 0.19, 0.42 ± 0.19, 0.28 ± 0.14, 0.31 ± 0.18, 0.37 ± 0.17, and 0.36 ± 0.17. In other words, based on the analysis of the joint PIEZO1–PIEZO2 interactome, proteins in the red and green clusters show a trend of being a bit less disordered compared to the average human protein, although this difference is not statistically significant. This finding suggests that while these PIEZO interactors may have slightly different structural characteristics than the overall human proteome, they do not constitute a heavily ordered or highly disordered subset based on this metric alone.

Liquid–liquid phase separation (LLPS) analysis of the PIEZO1/PIEZO2 interactome showed similar LLPS propensity between the green and red clusters, with nearly identical mean LLPS probabilities (0.480 and 0.483, respectively; Wilcoxon rank-sum test, *p* = 0.806). High-LLPS proteins, defined as LLPS probability ≥ 0.60, were also similarly distributed between clusters, with 18 proteins in the green cluster and 19 in the red cluster (Fisher’s exact test, *p* = 1.0). The similar LLPS profiles across the red and green STRING clusters suggest that LLPS propensity is a broader feature of the PIEZO-associated interactome rather than being restricted to one interaction cluster.

### 3.4. ClinVar Variant Distribution in PIEZO Channels

Clinically annotated variants for PIEZO1 and PIEZO2 were retrieved from ClinVar and summarized based on germline classification. For PIEZO1, a total of 1512 variants were identified, the majority of which were classified as variants of uncertain significance (VUS) or conflicting interpretations (*n* = 1382). Among variants with clearer clinical interpretation, 108 were classified as benign or likely benign, while 22 were classified as pathogenic or likely pathogenic. This distribution indicates a predominance of uncertain or inconsistently interpreted variants within PIEZO1. In contrast, PIEZO2 exhibited a smaller and more balanced variant dataset. Among variants with definitive classification, 47 were categorized as benign and 24 as pathogenic. Compared to PIEZO1, PIEZO2 shows a relatively higher proportion of pathogenic variants among classified entries, suggesting either increased clinical relevance or differences in the extent of characterization between the two proteins. Across both proteins, the predominance of VUS and conflicting classifications highlights the current limitations in functional interpretation of PIEZO channel variants. This is consistent with the large size, complex structure, and multifaceted functional roles of PIEZO proteins.

Overall, while both PIEZO1 and PIEZO2 have numerous clinically reported variants, PIEZO1 is characterized by a substantially larger pool of uncertain variants, whereas PIEZO2 exhibits a relatively higher proportion of variants with defined clinical significance.

### 3.5. Mapping of Variants to Intrinsically Disordered and Binding-Prone Regions

**Figure 7:**
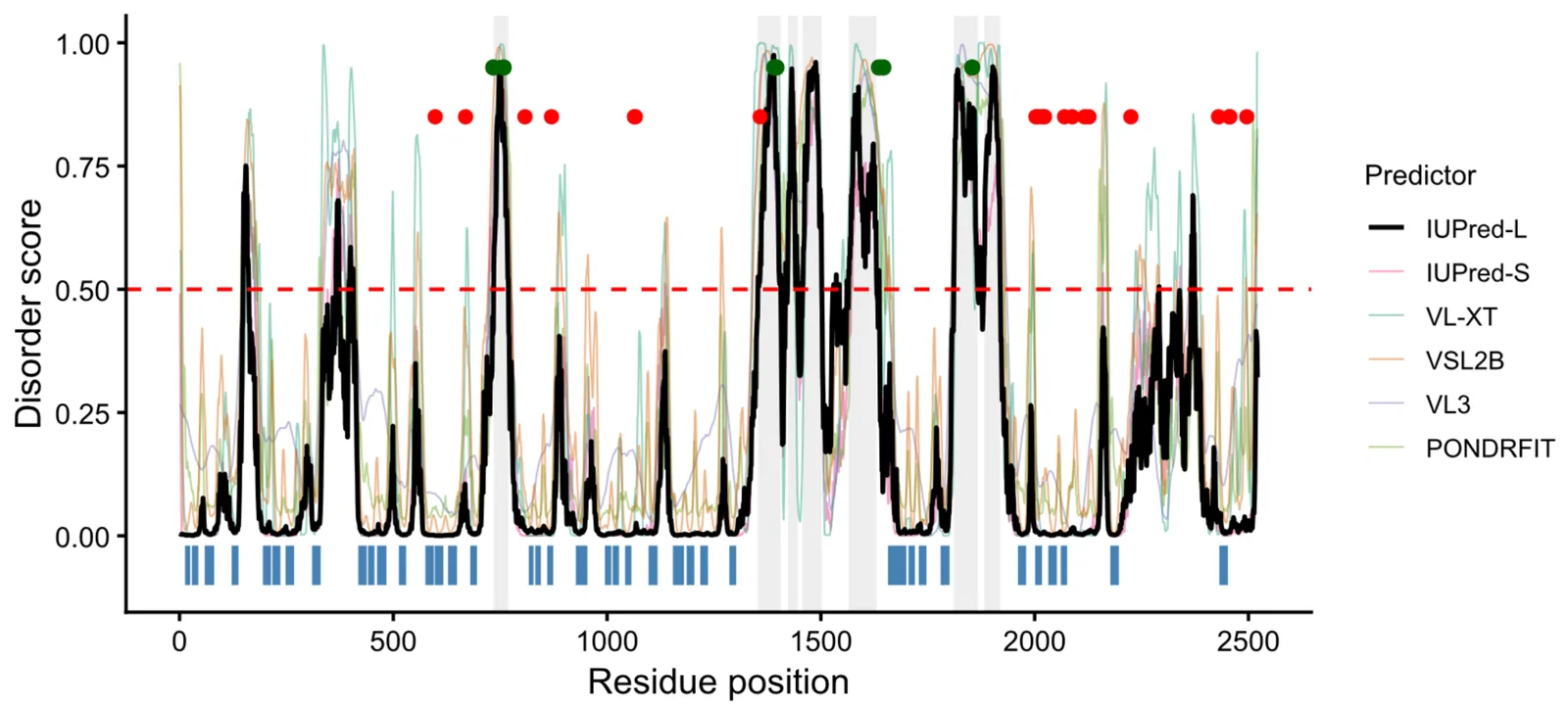
Intrinsic disorder landscape of human PIEZO1 across the full amino acid sequence using multiple disorder predictors. The black trace represents residue-level disorder propensity scores from IUPred-L, while colored traces indicate additional predictor models, with the red dashed line indicating the conventional disorder threshold (0.5). Grey shaded regions denote predicted intrinsically disordered regions (IDRs), blue bars indicate annotated transmembrane helices, green points mark UniProt-annotated post-translational modification sites, and red points indicate mapped disease-associated mutations. Disorder peaks are concentrated primarily in non-transmembrane regions, including several cytoplasmic loop segments.

**Figure 8:**
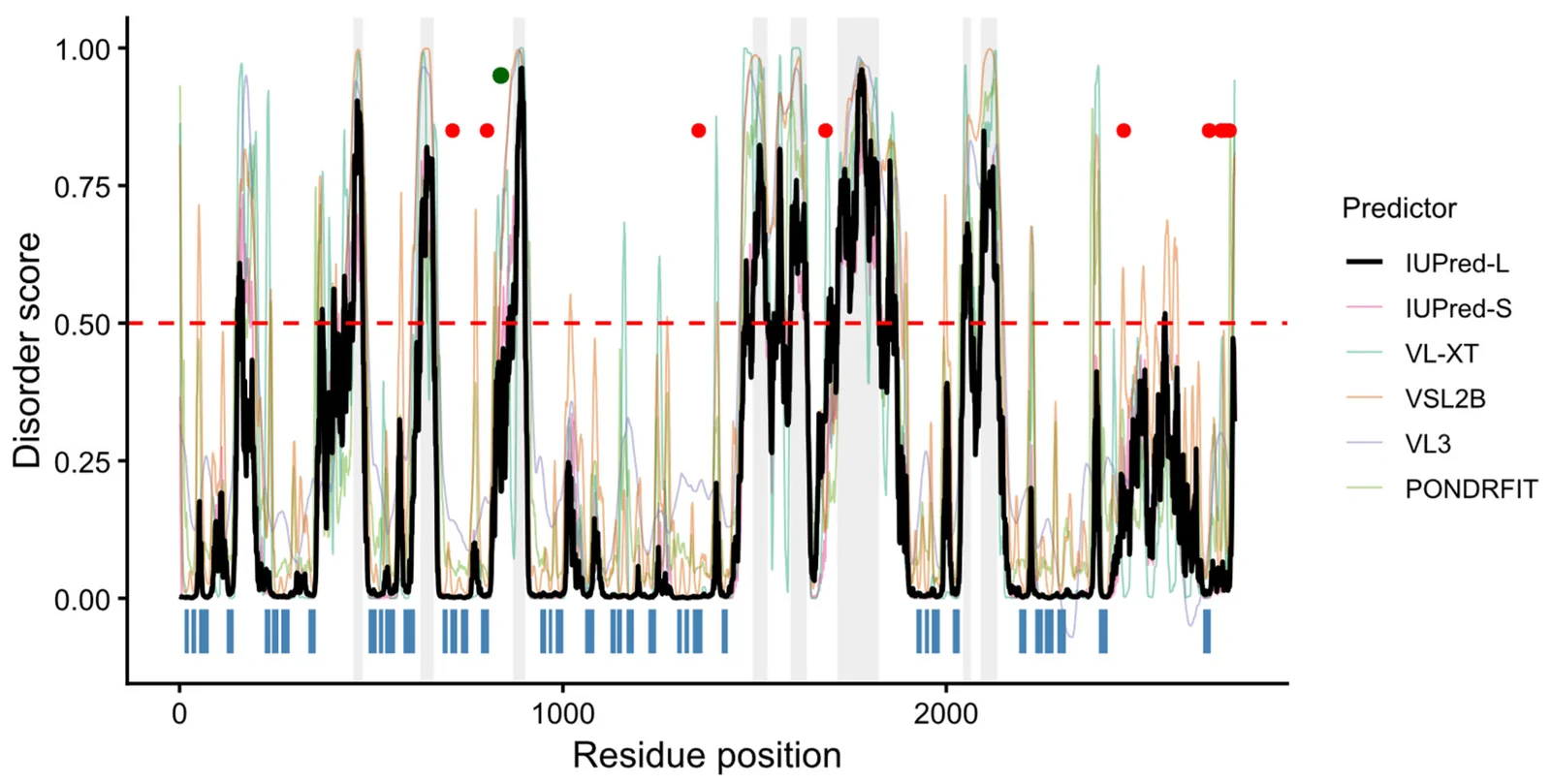
Intrinsic disorder landscape of human PIEZO2 across the full amino acid sequence using multiple disorder predictors. The black trace represents residue-level disorder propensity scores from IUPred-L, while colored traces indicate additional predictor models, with the red dashed line indicating the conventional disorder threshold (0.5). Grey shaded regions denote predicted intrinsically disordered regions (IDRs), blue bars indicate annotated transmembrane helices, green points mark UniProt-annotated post-translational modification sites, and red points indicate mapped disease-associated mutations. Higher disorder propensity is concentrated primarily in non-transmembrane regions, with multiple peaks in central and intracellular loop domains.

To assess the structural and functional context of genetic variation, ClinVar-derived variants were mapped onto predicted intrinsically disordered regions (IDRs) and disorder-mediated binding regions in PIEZO1 and PIEZO2. Residue-level disorder profiles, transmembrane regions, post-translational modifications (PTMs), and variant positions are visualized in Figure 7 (PIEZO1) and Figure 8 (PIEZO2).

In PIEZO1, benign variants were more frequently located within IDRs (23.1%) compared to pathogenic variants (4.5%), with most pathogenic variants (95.5%) occurring in ordered regions. Our statistical analysis showed a trend toward fewer pathogenic variants in disordered regions (Fisher’s exact test, *p* = 0.075; odds ratio = 0.16), although this did not reach statistical significance. Similarly, in PIEZO2, only ~4.2% of pathogenic variants were located within IDRs compared to ~10.6% of benign variants (*p* = 0.656), indicating a consistent but statistically weak trend in both proteins.

These patterns were visually shown by the disorder landscapes (Figure 7 and Figure 8), where variants (red points) are predominantly distributed within ordered regions below the disorder threshold, while intrinsically disordered regions (grey shaded areas) contain relatively few pathogenic variants. This suggests that the disruption of ordered domains may be more commonly associated with disease-associated phenotypes, whereas intrinsically disordered regions may be more tolerant or resilient to pathogenic mutations due to their flexible structural nature.

To test whether pathogenic variants occur in lower-disorder local sequence contexts, PONDR^®^ VSL2 scores were compared between benign and pathogenic PIEZO1 variants. Pathogenic variants had lower PONDR^®^ VSL2 scores than benign variants at the exact mutation site, with mean scores of 0.178 and 0.483, respectively, and this difference was significant by Wilcoxon rank-sum test (*p* = 0.0004). The same pattern was observed across the ±3-residue window, where pathogenic variants had a mean PONDR^®^ VSL2 score of 0.179 compared with 0.484 for benign variants (*p* = 0.0003). In PIEZO2, pathogenic variants also had lower PONDR^®^ VSL2 scores than benign variants at the exact mutation site, with mean scores of 0.198 and 0.345, respectively, and this difference was significant by Wilcoxon rank-sum test (*p* = 0.025). The same pattern was observed across the ±3-residue window, where pathogenic variants had a mean PONDR^®^ VSL2 score of 0.202 compared with 0.342 for benign variants (*p* = 0.031). Together, these findings support the interpretation that pathogenic PIEZO variants are associated with lower-disorder local sequence contexts, which is consistent with greater structural constraint at disease-associated mutation sites.

Mapping of variants to ANCHOR2-predicted binding regions showed a similar pattern. In PIEZO1, 18.5% of benign variants overlapped predicted binding-prone regions, whereas only 4.5% of pathogenic variants occurred within these regions, again suggesting reduced disease-associated mutations in functionally flexible segments. Although this difference was not statistically significant (*p* = 0.123), the directional consistency with IDR mapping supports a broader trend.

Representative residue-level analysis further highlighted the functional relevance of these regions. In PIEZO1, a mutation at residue 1358 occurs within a region exhibiting both elevated disorder (IUPred-L > 0.5) and ANCHOR2 score (>0.5), indicating a disordered segment with binding potential. A similar observation was made in PIEZO2, where a mutation at residue 1738 lies within a highly disordered (IUPred-L = 0.627) and strongly binding-prone region (ANCHOR2 = 0.715). The co-localization of mutations within regions predicted to be both flexible and interaction-prone is significant because these segments often function as molecular recognition features that mediate transient regulatory interactions [33,111]. Unlike mutations in rigid structured domains, mutations in these regions may not destabilize global protein structure but instead change binding specificity, conformational dynamics, or regulatory signaling. In mechanosensitive channels such as PIEZO proteins, mutation of these dynamic interfaces could influence processes like channel gating, partner recruitment, trafficking, or downstream mechanotransduction pathways. These can all provide an alternative mechanism by which mutations may contribute to disease.

In addition to disease-associated variants, post-translational modifications in PIEZO1 were significantly enriched within IDRs (4 of 7 sites; *p* = 0.0066), further supporting the role of disordered regions in facilitating regulatory interactions. Although fewer PTMs were observed in PIEZO2, their spatial relationship with disorder followed a similar pattern.

Collectively, these results show that intrinsically disordered and binding-prone regions in PIEZO channels contribute to functional flexibility and interaction capacity, while pathogenic mutations are primarily localized to ordered regions where they are more likely to disrupt protein stability or core function. At the same time, the presence of some mutations within disordered binding regions suggests that variance of dynamic interaction interfaces may also contribute to disease in specific cases.

### 3.6. Structural Modeling of Mutation-Containing Peptide Regions

**Figure 9:**
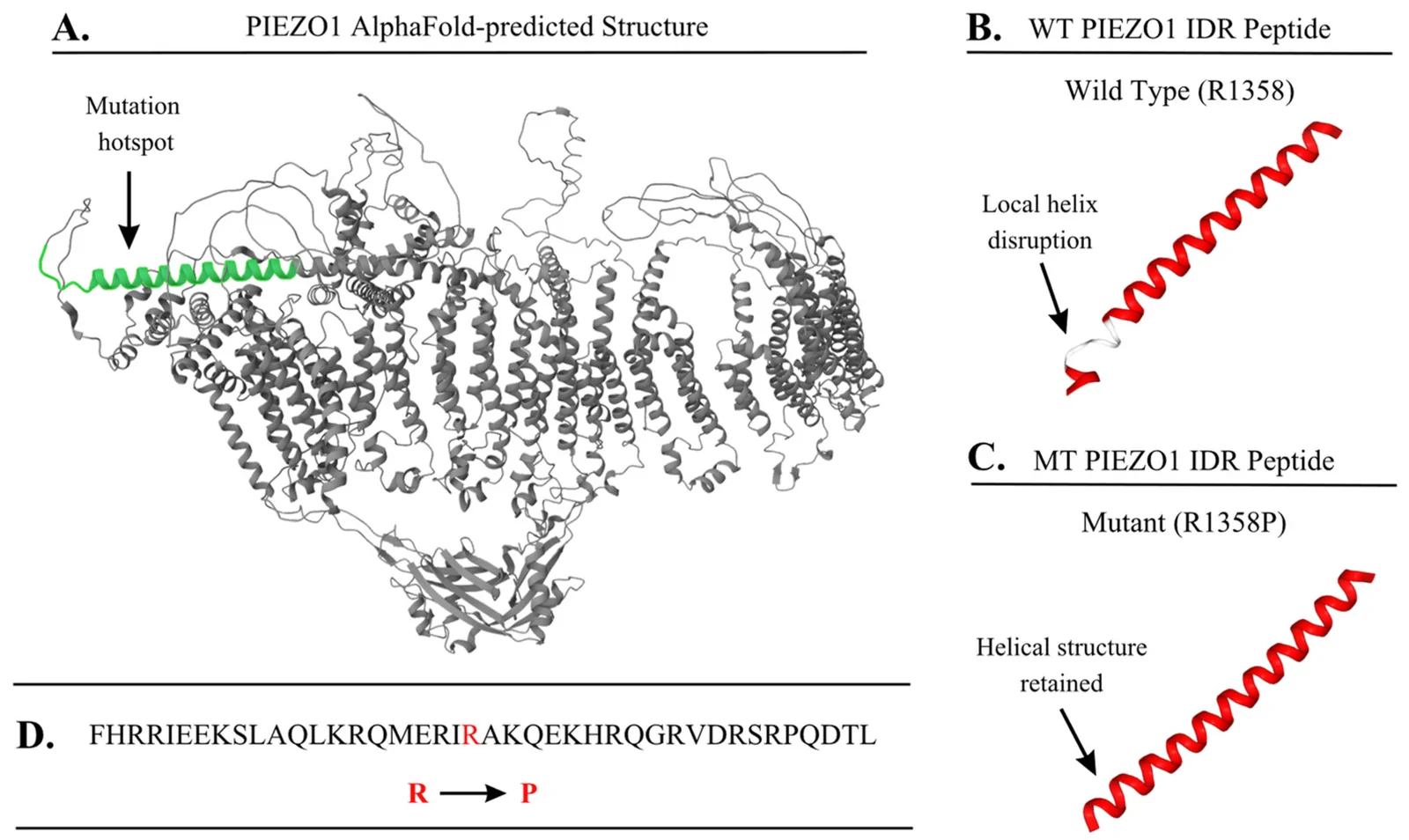
Structural modeling of the PIEZO1 R1358P variant within an intrinsically disordered hotspot region. (**A**) AlphaFold model of human PIEZO1 with the peptide region surrounding residue 1358 highlighted in green, indicating the mutation hotspot selected for local structural modeling. (**B**) PEP-FOLD prediction of the wild-type PIEZO1 peptide containing residue R1358, showing a local helix disruption near the N-terminal region. (**C**) Predicted structure of the mutant peptide (R1358P), showing retention of a continuous helical conformation. (**D**) Amino acid sequence of the modeled region with the R1358P substitution indicated in red.

**Figure 10:**
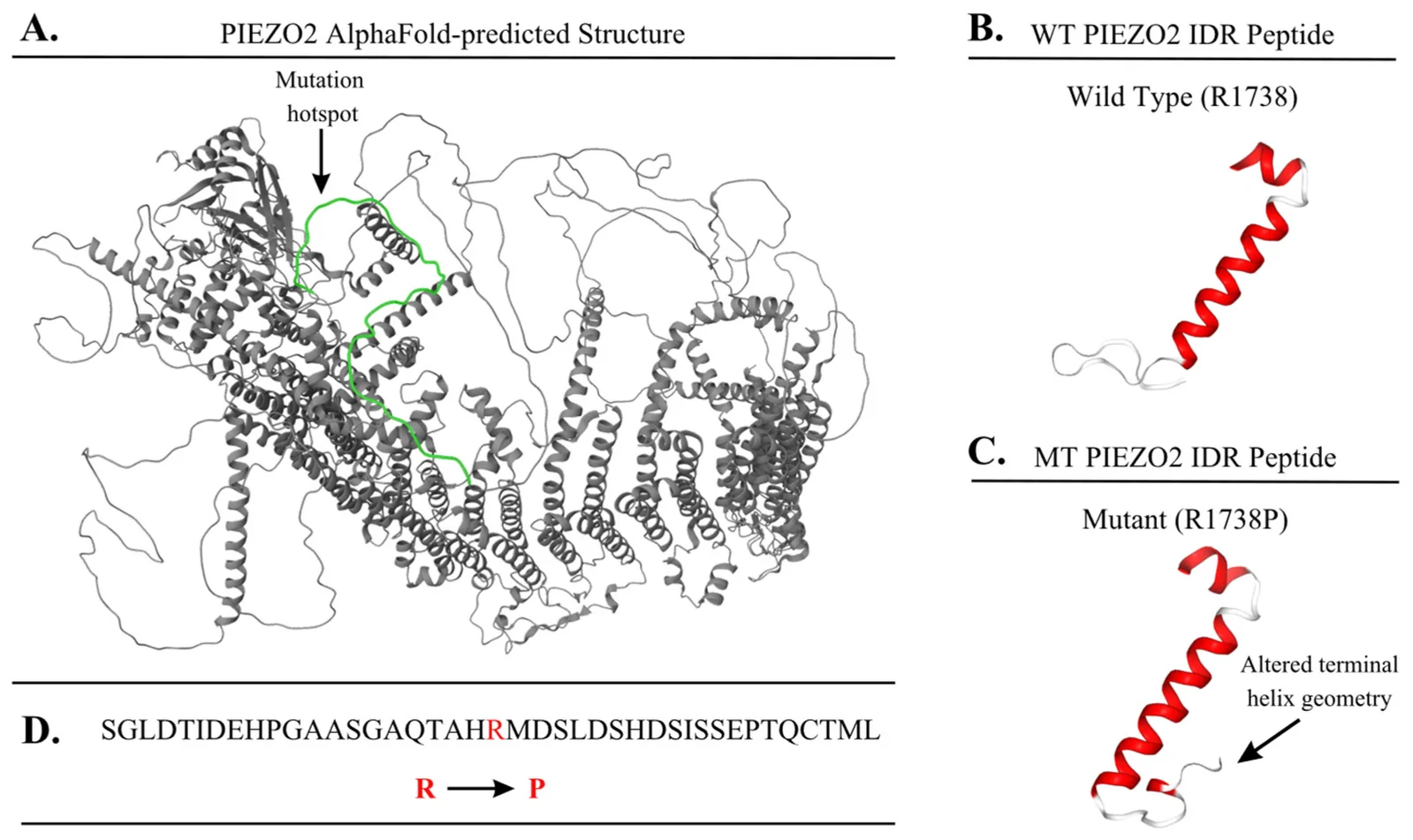
Structural modeling of the PIEZO2 R1738P variant within a predicted intrinsically disordered hotspot region. (**A**) AlphaFold model of human PIEZO2 with the peptide region surrounding residue 1738 highlighted in green, indicating the mutation hotspot selected for local structural modeling. (**B**) PEP-FOLD prediction of the wild-type PIEZO2 peptide containing residue R1738, showing a predominant central α-helical segment flanked by flexible terminal regions. (**C**) Predicted structure of the mutant peptide (R1738P), showing altered terminal helix geometry relative to the wild-type peptide. (**D**) Amino acid sequence of the modeled region with the R1738P substitution indicated in red.

Based on the analysis of intrinsic disorder, ANCHOR-predicted binding regions, and variant mapping, representative mutations located within disordered, binding-prone regions were selected for structural modeling. Specifically, residue 1358 in PIEZO1 and residue 1738 in PIEZO2 were chosen due to their localization within regions with both high disorder propensity and ANCHOR2 scores. To evaluate the structural impact of these mutations, we modeled peptide segments encompassing the selected residues using PEP-FOLD4. The predicted structures for wild-type and mutant sequences are shown in Figure 9 and Figure 10.

For PIEZO1, the comparison of predicted peptide structures showed a clear change in secondary structure between wild-type and mutant sequences. The wild-type peptide had a partial disruption of α-helical structure at the N-terminal region, transitioning into a more disordered conformation. In contrast, the mutant peptide displayed a more continuous and uniform α-helical structure across the same region. This suggests an increase in the local structural order and helical propensity associated with the mutation.

In PIEZO2, structural differences between wild-type and mutant peptides were more subtle. The wild-type peptide exhibited extended disordered regions, particularly at the N-terminus, alongside a central α-helical segment. The mutant peptide also had this overall structure but showed a reduction in disordered regions and a slightly more compact conformation. Unlike PIEZO1, however, the mutation did not result in a fully continuous helical structure, indicating that intrinsic flexibility was largely preserved.

These observations are consistent with the behavior of intrinsically disordered regions, which exist as dynamic conformational ensembles rather than fixed structures [33]. So, mutations within such regions are more likely to alter conformational preferences or interaction potential rather than cause stable structural rearrangements. The more pronounced structural change observed in PIEZO1 suggests a greater sensitivity of this region to mutation compared to PIEZO2. Overall, these results suggest that mutations within disordered, binding-prone regions may alter local structure and potentially influence interaction interfaces.

### 3.7. Evolutionary Conservation of PIEZO1 and PIEZO2

**Figure 11:**
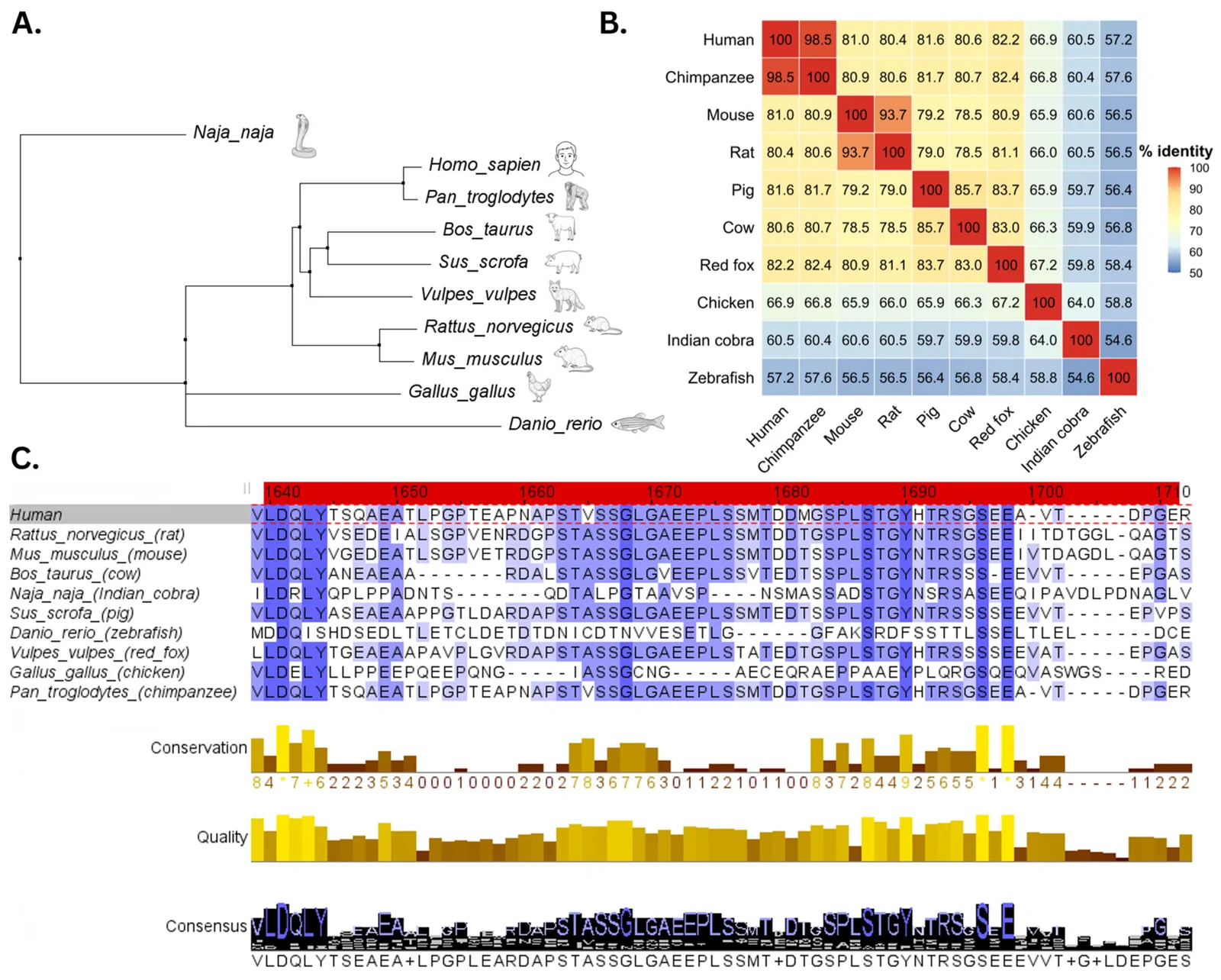
Evolutionary conservation of PIEZO1 and its major intrinsically disordered region. (**A**) Neighbor-joining tree of full-length PIEZO1 orthologs generated in Jalview from a Clustal Omega alignment using the BLOSUM62 matrix. Mammalian orthologs clustered together, with human and chimpanzee forming the closest pair, while chicken, Indian cobra, and zebrafish branched more distantly. (**B**) Pairwise percent identity heatmap of full-length PIEZO1 orthologs. Warmer colors indicate higher sequence identity, while cooler colors indicate lower identity, showing strong conservation among mammalian sequences and reduced identity in more distant vertebrates. (**C**) Multiple-sequence alignment of the major human PIEZO1 IDR region, corresponding to residues 1565–1630, highlighted in red above the alignment. Blue shading indicates percent identity at each aligned position, with darker shading representing stronger residue conservation. The conservation track summarizes residue conservation across the alignment, the quality track reflects alignment confidence/physicochemical similarity at each position, and the consensus sequence logo shows the most represented residues at each alignment position. In the conservation row, an asterisk (*) indicates the maximum conservation score, while a plus sign (+) indicates a conservation score of 10.

**Figure 12:**
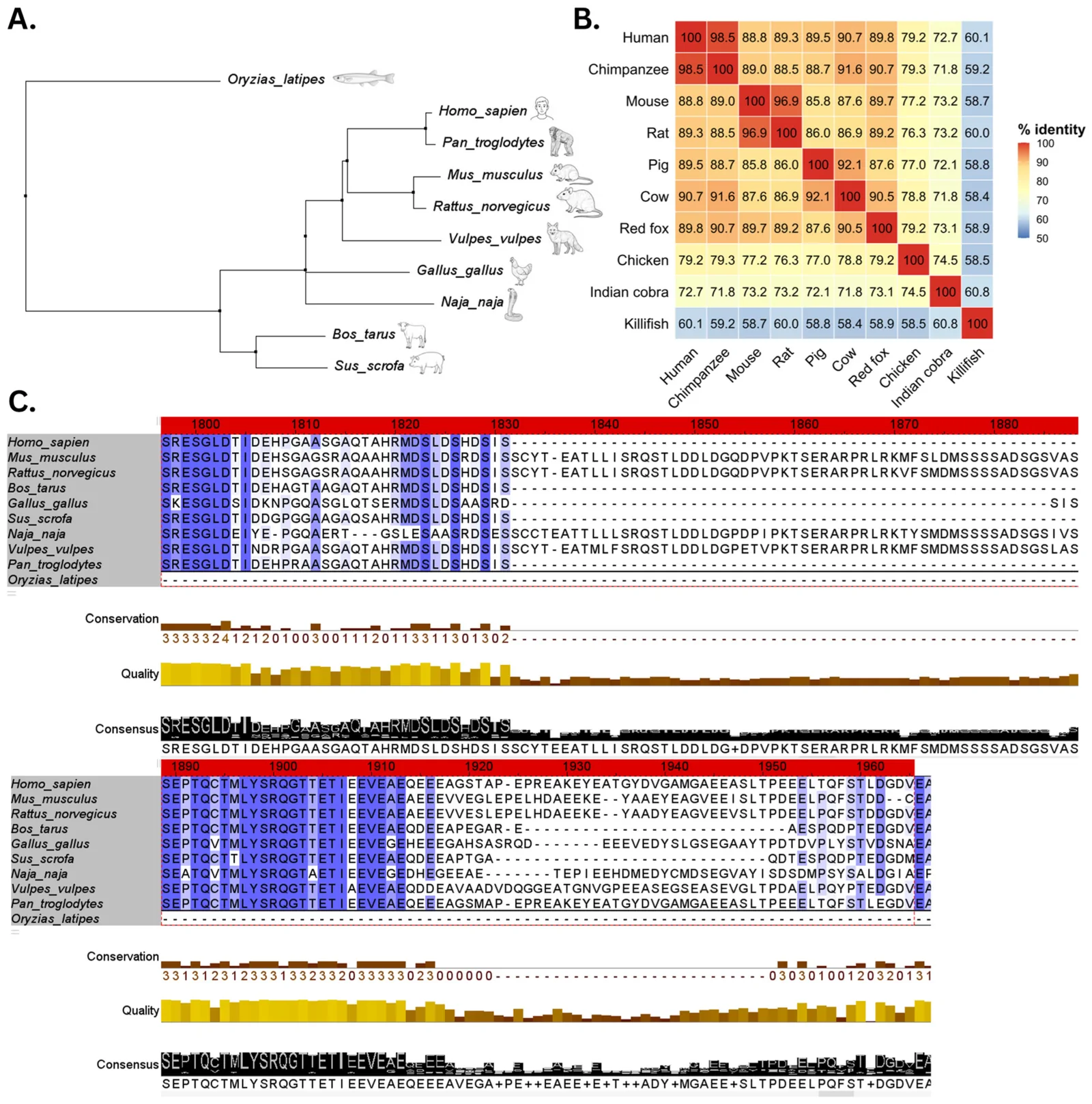
Evolutionary conservation of PIEZO2 and its major disordered region. (**A**) Neighbor-joining tree of full-length PIEZO2 orthologs generated in Jalview from a Clustal Omega alignment using the BLOSUM62 matrix. Mammalian orthologs clustered together, with human and chimpanzee forming the closest pair, while chicken, Indian cobra, and Japanese killifish branched more distantly. (**B**) Pairwise percent identity heatmap of full-length PIEZO2 orthologs. Warmer colors indicate higher sequence identity, while cooler colors indicate lower identity, showing strong conservation among mammalian sequences and reduced identity in more distant vertebrates. (**C**) Multiple-sequence alignment of the major PIEZO2 disordered region, highlighted in red above the alignment. Blue shading indicates percent identity at each aligned position, with darker shading representing stronger residue conservation. The conservation track summarizes residue conservation across the alignment, the quality track reflects alignment confidence and physicochemical similarity, and the consensus sequence logo shows the most represented residues at each aligned position. The major PIEZO2 disordered region showed uneven conservation, with short, conserved motif-like segments retained within a more variable, gap-rich, low-complexity region.

Clustal Omega alignments of full-length PIEZO1 and PIEZO2 orthologs generated in Jalview showed strong evolutionary conservation across vertebrates, especially among mammals. For PIEZO1, human PIEZO1 shared 98.53% identity with chimpanzee, while red fox, pig, mouse, bovine, and rat were all approximately 80–82% identical to the human sequence (Figure 11B). More distant vertebrates showed lower full-length identity, including chicken, Indian cobra, and zebrafish. A similar pattern was observed for PIEZO2, where human PIEZO2 shared 98.49% identity with chimpanzee, while bovine, red fox, pig, rat, and mouse were all approximately 89–91% identical to the human sequence (Figure 12B). More distant PIEZO2 orthologs showed lower full-length identity, including chicken, Indian cobra, and Japanese killifish. These patterns were also found in the neighbor-joining trees, where mammalian orthologs clustered together, human and chimpanzee formed the closest pair, and non-mammalian vertebrates branched more distantly (Figure 11A and Figure 12A). Together, these results indicate that both PIEZO1 and PIEZO2 are broadly conserved across vertebrates, consistent with strong evolutionary constraint on large mechanosensitive ion channels involved in membrane architecture, ion conduction, and mechanotransduction.

When conservation within the group of 10 orthologs was examined within the major predicted disordered regions, sequence identity was generally lower than for the full-length proteins. For PIEZO1, the major IDR spanning residues 1565–1630 was almost identical between human and chimpanzee but showed reduced identity across other mammals and a sharper decline in non-mammalian vertebrates (Figure 11C). For example, human and chicken PIEZO1 shared 66.90% identity across the full protein, but only 28.79% across the IDR. Human and cobra showed a similar pattern, decreasing from 60.49% full-length identity to 34.85% IDR identity. The zebrafish comparison showed only 11.11% identity, although this aligned over a very short segment and is best interpreted as poor local conservation rather than a clean full-region comparison. The PIEZO1 IDR alignment and conservation plot showed that residues 1565–1630 were not uniformly conserved at the primary-sequence level. Instead, this region contained short conserved or compositionally similar patches near the LDQLY motif, the serine/glycine/glutamate-rich central region, and the PLSTGY/HTRSGSEE-like region, surrounded by substitutions, gaps, and species-specific insertions or deletions.

For PIEZO2, the major disordered region spanning residues 1718–1824 showed a similar but more pronounced pattern of uneven conservation (Figure 12C). The Jalview alignment showed short conserved sequence blocks embedded within a more variable and gap-rich, low-complexity region. Conserved features were most apparent around the SRESGLDT region, the HRMDSLDSHDSIS-like segment, and the SEPTQCTMLYSRQGTTETI-like region. However, these conserved patches were interrupted by substitutions, gaps, and species-specific insertions or deletions, especially among more distant vertebrates. The consensus signal remained visible in parts of this region, even where conservation was low, suggesting that some orthologs retain similar disorder-associated residue composition rather than strict residue-by-residue conservation.

Importantly, IUPred predicted disorder in the corresponding aligned regions of several orthologs, even when exact residues were not strongly conserved. This suggests that the disordered character of these segments may be more conserved than the precise amino acid sequence. This pattern is biologically reasonable because IDRs often function through flexibility, charge distribution, short linear motifs, and overall amino acid composition rather than through a fixed folded structure. As a result, IDRs can tolerate substitutions and indels while still preserving disorder-prone properties or local interaction features.

ConSurf analysis was then used to evaluate residue-level conservation across broader sets of 150 selected orthologs for each protein. Across the full-length protein, PIEZO1 showed moderate-to-high conservation, with a mean ConSurf grade of 6.10, median grade of 6.0, and mean dominant amino acid percentage of 64.06%. Non-IDR residues were more conserved, with a mean grade of 6.58, median grade of 7.0, and mean dominant amino acid percentage of 67.64%. In contrast, the 15 high-confidence PIEZO1 IDRs identified by ≥75% predictor agreement had a much lower mean ConSurf grade of 3.09, median grade of 3.0, and mean dominant amino acid percentage of 41.70%. The major PIEZO1 IDR showed a similar pattern, with a mean ConSurf grade of 3.15, median grade of 3.0, and mean dominant amino acid percentage of 38.94%.

PIEZO2 showed the same overall trend. Across the full-length protein, PIEZO2 had a mean ConSurf grade of 5.96, median grade of 6.0, and mean dominant amino acid percentage of 62.33%. Non-IDR residues were more conserved, with a mean grade of 6.50, median grade of 7.0, and mean dominant amino acid percentage of 65.64%. In contrast, the 24 high-confidence PIEZO2 IDRs had a lower mean ConSurf grade of 2.78, median grade of 3.0, and mean dominant amino acid percentage of 42.84%. The major PIEZO2 disordered region spanning residues 1718–1824 showed the lowest conservation, with a mean ConSurf grade of 2.28, median grade of 1.0, and mean dominant amino acid percentage of 39.28% (Table 2).

Overall, these analyses support a shared two-level conservation pattern for PIEZO1 and PIEZO2. The full-length proteins are strongly conserved, especially among mammals, while predicted disordered regions are less conserved and more evolutionarily flexible. However, these regions are not random or completely unconstrained. This pattern suggests that PIEZO IDRs are under a different form of evolutionary constraint, where disorder propensity, amino acid composition, charge distribution, and short motif-like regions may be preserved even when exact residue identity is not. Rather than indicating that these disordered regions are unimportant, their reduced primary-sequence conservation may reflect the evolutionary flexibility expected of functional disordered regions.

## 4. Conclusions

Our study provides an analysis of intrinsic disorder, liquid–liquid phase separation, binding sites, evolutionary conservation, interaction networks, and clinically relevant mutations in the mechanosensitive ion channels PIEZO1 and PIEZO2. Both proteins exhibited moderate levels of intrinsic disorder, with PIEZO2 showing slightly greater disorder than PIEZO1. These disordered regions overlapped predicted binding-prone segments, suggesting roles in regulatory interactions and conformational flexibility.

Evolutionary conservation analyses showed that both PIEZO1 and PIEZO2 are strongly conserved across vertebrates, especially among mammals. However, their predicted IDRs were less conserved than non-IDR regions at the primary-sequence level. ConSurf analysis using 150 orthologs for each protein supported this pattern, with non-IDR residues showing higher conservation than high-confidence IDRs. This reduced residue-level conservation is consistent with the biology of IDRs, which often preserve flexibility, charge distribution, short motifs, and disorder-prone composition rather than exact amino acid sequence.

A key finding was that pathogenic variants were concentrated primarily in ordered regions of both proteins, whereas intrinsically disordered and binding-prone regions contained proportionally more benign variants and relatively few pathogenic mutations. This suggests that structurally constrained regions are less tolerant to mutation, while disordered regions may buffer sequence variation. Our modeling analysis also showed that selected mutations within disordered regions were predicted to alter local structural tendencies, indicating that the disruption of dynamic interaction interfaces may also contribute to disease. Overall, these findings highlight intrinsic disorder as an important component of PIEZO channel biology and provide a useful framework for understanding PIEZO-associated mutations and channelopathies.

There are several limitations to this study. First, the work is primarily computational. Therefore, our analyses depend on the assumptions and accuracy of the prediction algorithms used. Although we included multiple independent predictors to reduce tool-specific bias, the results require experimental validation. Furthermore, the predicted IDRs should not be interpreted as definitive structural assignments under physiological conditions, since their behavior may vary with pH, temperature, membrane environment, cell type, and interactions with auxiliary subunits, such as MDFI, MDFIC, and MDFIC2. Second, the peptide modeling and local disorder analyses suggest how selected variants may alter conformational tendencies. However, they do not directly measure channel gating, phase separation, or cellular function. Future experimental work using mutagenesis, biophysical assays, and functional channel measurements is required to validate the proposed roles of IDRs in PIEZO regulation and disease.

## Figures and Tables

**Table 1:** Percent predicted intrinsically disordered residues (PPIDRs) and average disorder score (ADS) for human PIEZO1 and PIEZO2 evaluated by seven disorder prediction algorithms compiled from RIDAO.

	PIEZO1		PIEZO2	
Disorder Predictor	PPIDR (%)	ADS	PPIDR (%)	ADS
PONDR^®^ VLXT	26.89	0.2750	24.78	0.2605
PONDR^®^ VSL2	27.13	0.3282	31.69	0.3608
PONDR^®^ VL3	22.45	0.3142	26.02	0.3482
PONDR^®^ FIT	20.15	0.2664	22.27	0.2824
IUPred-Short	17.37	0.1928	19.22	0.1984
IUPred-Long	14.76	0.1884	15.37	0.1965
Mean Disorder Profile	20.31	0.2608	23.76	0.2745

**Table 2:** ConSurf conservation summary for full-length PIEZO1/PIEZO2 and high-confidence intrinsically disordered regions. Residue-level conservation was calculated using ConSurf across 150 selected homologs for each protein. ConSurf grades range from 1 to 9, with lower values indicating more variable residues and higher values indicating more conserved residues. High-confidence IDRs were defined as regions where ≥75% of disorder predictors agreed. Mean dominant AA % represents the average percentage of homologs containing the most common amino acid at each position. The major region analyzed for PIEZO1 corresponded to residues 1565–1630, while the major disordered region analyzed for PIEZO2 corresponded to residues 1718–1824.

Protein	Region Analyzed	Mean ConSurf Grade	Median ConSurf Grade	Mean Dominant AA %
PIEZO1	Full protein	6.10	6.0	64.06%
	Non-IDR residues	6.58	7.0	67.64%
	All high-confidence IDRs	3.09	3.0	41.70%
	Major IDR (1565–1630)	3.15	3.0	38.94%
PIEZO2	Full protein	5.96	6.0	62.33%
	Non-IDR residues	6.50	7.0	65.64%
	All high-confidence IDRs	2.78	3.0	42.84%
	Major IDR (1718–1824)	2.28	1.0	39.28%

## Data Availability

Data are contained within the article.

## Abbreviations

The following abbreviations are used in this manuscript:

ADS	Average disorder score
ALS	Amyotrophic lateral sclerosis
ANCHOR	Disorder-based binding-region predictor
BAV	Bicuspid aortic valve
BC	Biomolecular condensate
BLOSUM62	Blocks Substitution Matrix 62
CDF	Cumulative distribution function
CH	Charge–hydropathy
ClinVar	Clinical Variant database
CNS	Central nervous system
ConSurf	Conservation Surface-Mapping tool
D2P2	Database of Disordered Protein Predictions
DOMS	Delayed-Onset Muscle Soreness
DPR	Droplet-promoting region
FDR	False discovery rate
FFL	Force-from-lipids
GLD	Generalized lymphatic dysplasia
GO	Gene Ontology
IDP	Intrinsically disordered protein
IDR	Intrinsically disordered region
IUPred	Intrinsically Unstructured Protein predictor
KEGG	Kyoto Encyclopedia of Genes and Genomes
LLPS	Liquid–liquid phase separation
MAFFT	Multiple Alignment using Fast Fourier Transform
MDP	Mean disorder profile
MLO	Membrane-less organelle
MoRF	Molecular recognition feature
MS	Mass spectrometry
NMR	Nuclear magnetic resonance
PDB	Protein Data Bank
PEP-FOLD	Peptide folding prediction server
p_DP_	Residue-level droplet-promoting probability
p_LDDT_	Predicted local distance difference test
p_LLPS_	Probability of liquid–liquid phase separation
POIS	Post Orgasmic Illness Syndrome
PONDR	Predictor of Natural Disordered Regions
PPI	Protein–protein interaction
PPIDR	Percentage of predicted intrinsically disordered residues
PrDOS	Protein DisOrder prediction System
PTM	Post-translational modification
RCDI	Regions with context dependent interactions
RIDAO	Rapid Intrinsic Disorder Analysis Online
SCOP	Structural Classification of Proteins
sOPEP	Optimized potential for efficient structure prediction
STRING	Search Tool for the Retrieval of Interacting Genes/Proteins
UniProt	Universal Protein Resource
VUS	Variant of uncertain significance
